# Dynamical responses predict a distal site that modulates activity in an antibiotic resistance enzyme†

**DOI:** 10.1039/d4sc03295k

**Published:** 2024-09-30

**Authors:** Michael Beer, Ana Sofia F. Oliveira, Catherine L. Tooke, Philip Hinchliffe, Angie Tsz Yan Li, Balazs Balega, James Spencer, Adrian J. Mulholland

**Affiliations:** a School of Cellular and Molecular Medicine, University of Bristol Bristol BS8 1TD UK Jim.spencer@bristol.ac.uk; b Centre for Computational Chemistry, School of Chemistry, University of Bristol BS8 1TS UK Adrian.mulholland@bristol.ac.uk

## Abstract

β-Lactamases, which hydrolyse β-lactam antibiotics, are key determinants of antibiotic resistance. Predicting the sites and effects of distal mutations in enzymes is challenging. For β-lactamases, the ability to make such predictions would contribute to understanding activity against, and development of, antibiotics and inhibitors to combat resistance. Here, using dynamical non-equilibrium molecular dynamics (D-NEMD) simulations combined with experiments, we demonstrate that intramolecular communication networks differ in three class A SulpHydryl Variant (SHV)-type β-lactamases. Differences in network architecture and correlated motions link to catalytic efficiency and β-lactam substrate spectrum. Further, the simulations identify a distal residue at position 89 in the clinically important *Klebsiella pneumoniae* carbapenemase 2 (KPC-2), as a participant in similar networks, suggesting that mutation at this position would modulate enzyme activity. Experimental kinetic, biophysical and structural characterisation of the naturally occurring, but previously biochemically uncharacterised, KPC-2^G89D^ mutant with several antibiotics and inhibitors reveals significant changes in hydrolytic spectrum, specifically reducing activity towards carbapenems without effecting major structural or stability changes. These results show that D-NEMD simulations can predict distal sites where mutation affects enzyme activity. This approach could have broad application in understanding enzyme evolution, and in engineering of natural and *de novo* enzymes.

## Abstract 2

Dynamical non-equilibrium molecular dynamics (D-NEMD) simulations pinpoint distal sites in class A β-lactamases as targets for mutation. Experiments show mutation at a predicted distal site changes the spectrum of enzyme activity against antibiotics.

## Graphical abstract

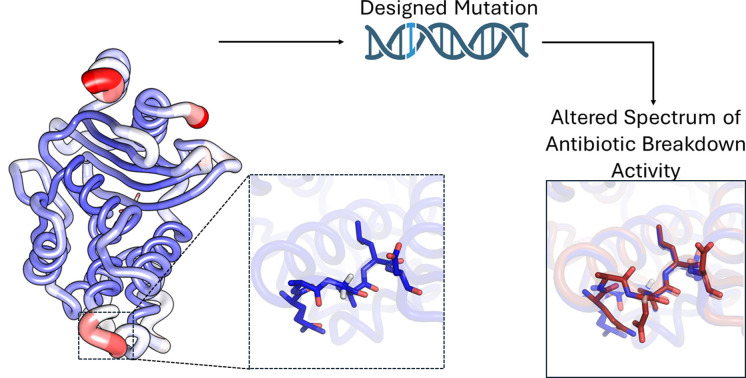

## Introduction

**Fig. 1:**
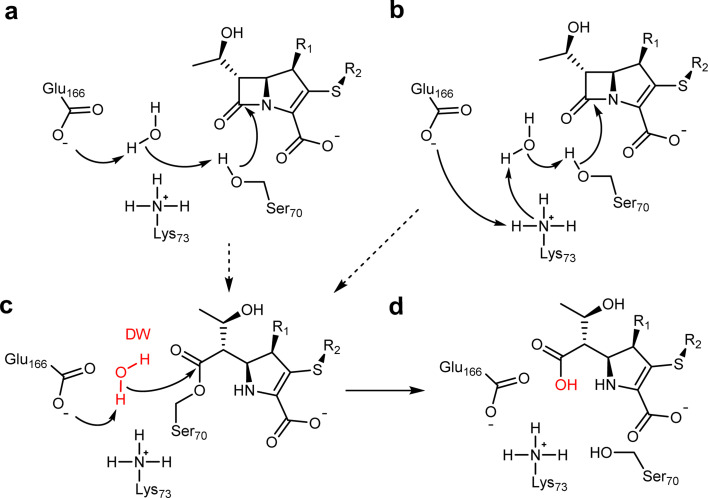
β-Lactam hydrolysis catalysed by class A β-lactamases. Substrate shown is a generalised carbapenem. Acylation (dashed arrows) catalysed by Glu166 (a) or Glu166/Lys73 (b) general bases forms the covalent acyl–enzyme (c). Deacylation requires nucleophilic attack of the deacylating water molecule (DW, red), activated by proton transfer to Glu166, on the acyl–enzyme carbonyl, to regenerate the enzyme and liberate the hydrolysed β-lactam (d).^3^

Antimicrobial resistance (AMR) is a growing global healthcare crisis, associated with 4.95 million deaths in 2019.^1^ β-Lactams account for approximately 65% of all antibiotic usage in humans worldwide^2^ and are vital components of our antibiotic arsenal. There are four major β-lactam classes: penicillins, cephalosporins (including oxyiminocephalosporins such as ceftazidime), carbapenems, and monobactams (Fig. S1†). In Gram-negative bacteria (such as *E. coli*), which are leading causes of antibiotic-resistant infections worldwide, the primary mechanism of β-lactam resistance is the expression of β-lactamases,^2^ enzymes that hydrolyse the β-lactam ring to abolish antibacterial activity^3^ (Fig. 1). There are four classes (A–D) of β-lactamases, with class A being the largest and most widely disseminated.^3^ This includes numerous enzyme groups, including the widely distributed SHV (SulpHydryl Variant) and KPC (*Klebsiella pneumoniae* carbapenemase) families, and collectively has activity against all clinically used β-lactam antibiotics.^4^ Single and multiple amino acid substitutions expand the β-lactamase spectrum of activity to cover new β-lactams and/or reduce susceptibility to β-lactamase inhibitors that are co-administered with β-lactams to treat resistant infections.^5^ While, in some cases, it is clear that individual mutations exert their effects by altering active site structure,^6^ others are situated far from the active site and the reasons for their (often profound) effects upon activity are obscure. Understanding and predicting the effects of β-lactamase mutations will inform more effective β-lactam use and drive the development both of new β-lactam antibiotics and of more effective β-lactamase inhibitors.

Remote mutations are well known to influence the behaviour of both natural and designed enzymes,^7,8^ yet understanding of structure and function is still largely concentrated on active sites and binding interfaces. Understanding the roles of residues in distal regions will expand the scope of rational protein design.^8^ Furthermore, the ability to effectively predict and identify the impact of mutations remote from active sites and ligand/protein binding regions should aid therapeutic development for multiple clinical pathologies *e.g.* cancer,^9^ HIV/AIDS^10,11^ and mental health disorders.^12^ There have been previous attempts to employ path calculation methods to interrogate the conformational ensembles of proteins and also to predict residues key to catalysis, through the analysis of equilibrium simulations or static structures.^13–15^ Here, we employ dynamical non-equilibrium molecular dynamics (D-NEMD) simulations, an emerging computational technique that applies the Kubo–Onsager relation^16,17^ to measure the linear response of a protein to a perturbation that pushes the system out of equilibrium. These simulations can reveal allosteric communication networks within proteins.^18–22^ In the D-NEMD approach, an external perturbation (*e.g.* deletion of a bound ligand) is applied to an equilibrium simulation. This enables the response of the protein to the perturbation to be directly measured by comparing the equilibrium trajectory with multiple parallel non-equilibrium simulations (Fig. S2†).^18^ This conceptually simple but powerful approach enables computing of the time-dependent dynamic response of the protein, with assessment of statistical significance. D-NEMD simulations have recently been applied to identify structural communication pathways in a range of biomolecular systems.^18–22^ D-NEMD simulations of the β-lactamase enzymes KPC-2 and TEM-1 previously deleted an allosterically bound ligand as the applied perturbation, identifying a network of residues that link the allosteric site to the active site.^23^ Here, we apply D-NEMD to show that such networks differ between point variants of the SHV β-lactamase with diverse activities towards different β-lactam substrates; and to identify a site in the KPC-2 β-lactamase where we would predict mutation to affect activity through communication with the active site. Using steady- and pre-steady state kinetics, circular dichroism spectroscopy and high-resolution X-ray crystallography, we reveal that a single predicted mutation, situated within a distal loop, significantly impacts the KPC-2 activity spectrum. This work highlights the effectiveness of D-NEMD simulations as a method to identify previously uncharacterised mutations, distant from enzyme active sites, that affect activity.

## Experimental methods

### Model set up

For SHV-1, the crystal structure of the S70C mutant bound to sulbactam (PDB ID 4FH2 (ref. 24)) was used with the Cys70 residue mutated back to Ser70 in WinCoot 0.9.8.1 and modelled into the Serine 70 electron density from the uncomplexed structure of SHV-1 (PDB ID 1SHV^25^). The SHV-2 and SHV-38 structures were generated using AlphaFold Colab^26^ and the sulbactam structure modelled into the active site region, using the electron density from the structure of the SHV-1 S70C complex. Protonation states were assigned using the PropKa program at pH 7.4.^27^

The KPC-2^G89D^ mutant structure was created using AlphaFold Colab,^26^ and the compound 2 ligand modelled into the active site using the electron density map of the compound 2 : KPC-2 complex (PDB ID 6D16 (ref. 28)) in WinCoot 0.9.8.1.^29^

All complexes were set up for molecular dynamics simulations using GROMACS 2019.1 ^30^ using the Amber ff14SB forcefield^31^ for the protein and the GAFF parameters for the ligands.^32^ Ligand atoms were parameterised using the ACPYPE server^33^ and RESP charges calculated using the R. E. D. server.^34^ Each system was solvated using TIP3P^35^ water in a cubic box with 10 Å distance between the edge and the solute. Na^+^ and Cl^−^ counter ions to a concentration of 180 mM were added to neutralise the system. Each system was minimised by steepest descent for 10 000 steps, to relieve bad contacts. The system was initially equilibrated in the *NVT* ensemble over 500 ps with restraints on all heavy atoms (force constant of 1000 kJ mol^−1^ nm^−1^), using *V*-rescale temperature coupling. Two coupling groups were used (protein and ligand, water and ions) with a coupling constant of 0.05 ps. The next step of equilibration was performed for 500 ps in the *NPT* ensemble using Berendsen pressure coupling, *V*-rescale temperature coupling (same two coupling groups as *NVT* equilibration but with a coupling constant of 0.1 ps). All Cα atoms and ligand heavy atoms were restrained in this equilibration stage with a force constant of 1000 kJ mol^−1^ nm^−1^. A second 500 ps *NPT* ensemble equilibration was then performed using Berendsen pressure coupling^36^ with restraints only on ligand heavy atoms (100 kJ mol^−1^ nm^−1^ force constant) and the same *V*-rescale temperature coupling parameters as the previous *NPT* equilibration step. 250 ns production simulations were run in the *NPT* ensemble using Parrinello–Rahman pressure coupling^37^ and the same *V*-rescale temperature coupling parameters. In the production simulations, the neighbours list was updated every 40 steps. All hydrogen bonds were constrained to their equilibrium lengths with the LINCS algorithm, except for the water molecules, which were kept rigid with the SETTLE algorithm.

Five 250 ns equilibrium MD simulations were run in the GROMACS 2019.1 software package with each of the prepared SHV-1, SHV-2, SHV-38, KPC-2 and KPC-2^G89D^ systems.^30^ The simulations were considered fully equilibrated for the D-NEMD approach after 50 ns (Fig. S1 and S2†).

### Dynamical nonequilibrium MD (D-NEMD) simulations

To study signal propagation between the active site and the rest of the protein, 200, 5 ns long, dynamical nonequilibrium simulations were performed for SHV-1, SHV-2, SHV-38, KPC-2 and KPC-2^G89D^. These simulations drive, and allow for the characterisation of, rapid conformational changes in the system and permit mapping of the communication networks within the proteins using the Kubo–Onsager approach. At each 5 ns time point, from 50 ns to 250 ns, a structure file of the entire system was extracted, resulting in 40 conformations per replicate and thus 200 starting conformations per system. The ligand atoms (bound to the active site of each system) were then deleted. The resulting non-equilibrium system was run for 5 ns in the GROMACS 2019.1 software package in identical conditions to the 250 ns MD simulations described above. The ‘null perturbation’ was performed by randomising the velocities (maintaining a Boltzmann distribution) at each 5 ns time point, instead of deleting the ligand atoms. The resulting ‘null perturbed’ system was run for 5 ns in the GROMACS 2019.1 software package in identical conditions to the 250 ns original equilibrium simulation.

The distance between the two systems at equivalent time points (*e.g.* 55 ns on the equilibrium MD simulation, compared to the last frame of the 5 ns non-equilibrium MD trajectory obtained starting from the conformation extracted at the 50 ns time point on the equilibrium MD simulation) was calculated for each Cα in the protein. This was done for each system after 10 ps, 50 ps, 100 ps, 500 ps, 1 ns, 3 ns and 5 ns of simulation. Cα deviation values were averaged over all 200 non-equilibrium simulations per system and the standard deviations and standard error of the mean calculated. The Cα deviation plot for the 5 ns time points was used to identify communication networks in all systems. The statistical significance of differences between systems in Cα deviation of individual residues was calculated using the Student's *t*-test, with the cut-off set at *p* = 0.05. False discovery rate corrections were applied using the Benjamini and Hochberg method implemented in GraphPad Prism 9.3.1 (GraphPad Software, La Jolla, CA, USA; https://www.graphpad.com) and set at a 5% cut-off.^38^

### Protein expression and purification

G89D and E166Q mutations of KPC-2 were produced by site directed mutagenesis of the previously constructed pET28a-KPC-2 vector^39^ The primers used to generate the E166Q mutant were 5′-TCA GCT CCA GCT GCC AGC GGT CCA G-3′ and 5′-CTG GAC CGC TGG CAG CTG GAG CTG A-3′ and site directed mutagenesis was performed using the QuikChange II XL Lightning Site-Directed Mutagenesis Kit, following the manufacturer's instructions (Agilent Genomics). Proteins were subsequently expressed and purified as previously described.^40^

### Steady-state kinetics

Antibiotic hydrolysis was measured at 25 °C in kinetics buffer (10 mM HEPES, pH 7.5 and 150 mM NaCl, 100 μg per mL bovine serine albumin (BSA)). Steady-state kinetic parameters were calculated by measuring the hydrolysis of β-lactam antibiotics (ampicillin Δ*ε*_235_ = −900, cefotaxime Δ*ε*_262_ = −7660, ceftazidime Δ*ε*_265_ = −7445, meropenem Δ*ε*_297_ = −11 500).^39–42^ Hydrolysis was followed using Greiner half area 96-well plates and a BMG CLARIOstar Plus microplate reader. GraphPad Prism 9.3.1 (GraphPad Software, La Jolla, CA, USA; https://www.graphpad.com) was used to calculate kinetic parameters. Initial rates of antibiotic hydrolysis measured across a range of antibiotic concentrations were used to calculate steady-state parameters according to the Michaelis–Menten equation. The *k*_cat_/*K*_M_ value for cefotaxime was additionally calculated by fitting the complete hydrolysis curve using the following equation:*A*_*t*_ = *A*_∞_ + (*A*_0_ − *A*_∞_)e^−*kt*^

IC_50_ values were calculated by following the initial rate of nitrocefin hydrolysis (200 μM) at 486 nM (Δ*ε*_486_ = 20 500 M^−1^ cm^−1^),^39^ after a 10 minute pre-incubation of enzyme and inhibitor. Both inhibitors were dissolved in kinetics buffer.

### Pre-steady-state kinetics

Pre-steady state kinetics were measured by mixing meropenem with KPC-2 or KPC-2^G89D^ (both 10 nM concentration) in an Applied Photophysics SX20 stopped-flow spectrometer connected to a photodiode array detector in kinetics buffer. Data were fitted from 0.005 to 0.3 s for both 5 repeats of each of KPC-2 : meropenem and KPC-2^G89D^ : meropenem. *k*_1_ and *k*_−1_ were calculated from the gradient and the *y*-intercept of the *k*_obs_*vs.* substrate concentration curves. *K*_D_ is calculated using the following equation:
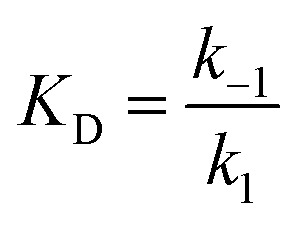


### Circular dichroism (CD)

Protein samples were prepared to a concentration of 20 μM in potassium phosphate buffer (100 mM, pH 7.6). The CD spectra were obtained using a JASCO J-1500 spectrophotometer. For thermal melt experiments, CD signal was measured at 220 nm between 5–95 °C, changing the temperature 1 °C per minute. A full CD spectrum at 25 °C for each protein was also recorded, measuring between 200–250 nm. Eight spectra were obtained for each protein and the results averaged to give the final spectra (Fig. S6†). Here, we report CD results as mean residual ellipticity (MRE) which was obtained from the raw data using the equation reported in Hutchins *et al.*^43^

### Crystallisation and ligand soaking

Crystals were grown at 20 °C using sitting drop vapor diffusion in CrysChem 24-well plates (Hampton Research), with wells equilibrated against 500 μL crystal buffer (5% (v/v) ethanol with 1.8–2.0 M NH_4_(SO_4_)_2_). Drops consisted of 1 μL of crystal seed (generated from crushed KPC-2 crystals), 2 μL protein (30 mg per mL aliquots), and 1 μL of crystallisation reagent.^40^

KPC-2^G89D^ crystals were soaked in mother liquor supplemented with 30 mM avibactam for 4 hours, before being brief exposure to mother liquor supplemented with 25% (v/v) glycerol and flash frozen in liquid nitrogen. For the meropenem and imipenem acylenzyme structures, crystals were soaked in mother liquor supplemented with 100 mM meropenem for 2.5 hours and 30 mM imipenem for 2.5 hours, respectively, before being briefly soaked in mother liquor with 25% (v/v) glycerol and flash frozen in liquid nitrogen.

### X-ray diffraction data collection and structural determination

Diffraction data were collected at Diamond Light Source on beamline I03 (Table S1†), using an Eiger2 XE 16M detector with an exposure time of 0.004 s per image.

In all cases the images were indexed and integrated using the Dials^44^ and Xia2 (ref. 45) processing pipelines at Diamond Light Source. Phases were calculated using Fourier transform in PHENIX^46^ with KPC-2^E166Q^ with the meropenem ligand removed (PDB ID 8AKL^47^) as the starting structure. The structure was completed with iterative rounds of refinement in PHENIX^46^ and manual model building in Wincoot.^29^ All ligand restraints were calculated using the Grade web server (https://grade.globalphasing.org/).

## Results and discussion

### Communication networks differ between single-point variants of SHV β-lactamases

**Fig. 2:**
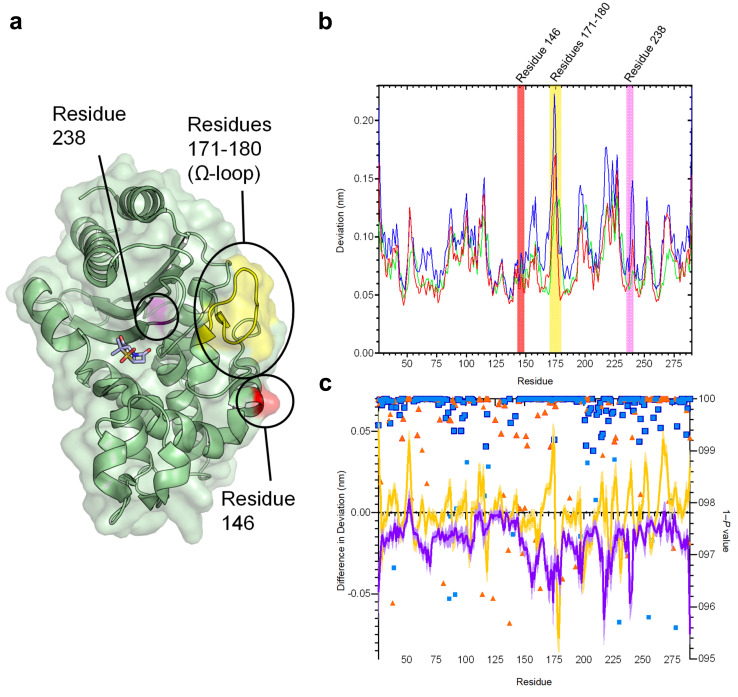
Residue deviations in SHV-1, -2 and -38 from D-NEMD simulations. (a) Cartoon of SHV-1 β-lactamase, showing positions of mutations in SHV-2 (SHV-1 G238S, pink) and SHV-38 (SHV-1 A146V, red), and part of the Ω-loop (residues 171–180, yellow) close to the active site. Sulbactam ligand is shown as sticks. (b) Per-residue deviations calculated using the Kubo–Onsager relation^16,17^ for SHV-1 (red), SHV-2 (blue) and SHV-38 (green). Residues 146 (red bar), 171–180 (yellow bar) and 238 (pink bar) are highlighted. Deviations are calculated by averaging Cα RMSD values between perturbed (*i.e.* after removal of bound sulbactam ligand) and unperturbed systems 5 ns after perturbation for each residue (c) differences in deviations 5 ns after perturbation, highlighting the effect of mutations on the communication network. Plots show SHV-1 *vs.* SHV-2 (purple) and SHV-1 *vs.* SHV-38 (yellow). Negative values indicate that SHV-2 or SHV-38 have greater Cα deviations than SHV-1 at specified residues. Error bars (one standard error of difference) are displayed above and below difference plots (lighter shading). Symbols show 1 − *P* values ≥0.95 (right axis, indicating statistical significance) for differences in deviation for SHV-1 *vs.* SHV-2 (blue squares) and SHV-1 *vs.* SHV-38 (orange triangles). Values highlighted with a navy (SHV-1 *vs.* SHV-2) or orange (SHV-2 *vs.* SHV-38) outline remain significant after false discovery rate corrections.

The SHV family of class A β-lactamases includes enzymes with broad-spectrum, extended-spectrum and carbapenemase activity.^48^ Broad spectrum β-lactamases efficiently hydrolyse penicillins, the most commonly prescribed antibiotics in the UK, and some early-generation cephalosporins.^49^ Extended-spectrum β-lactamases (ESBLs) further hydrolyse the later generation oxyiminocephalosporins, widely used antibiotics that are on the World Health Organization's list of essential medicines. Carbapenemases turn over carbapenem antibiotics, previously considered ‘last resort’ β-lactams. Many single-point variants of the broad-spectrum parent enzyme, SHV-1 (Fig. 2a) have altered activity profiles. For example, SHV-2 (SHV-1^G238S^) and SHV-38 (SHV-1^A146V^) have increased activity against oxyiminocephalosporins and carbapenems, classing them as extended-spectrum and carbapenemase enzymes, respectively (Table S1†).^48,50^ We used D-NEMD simulations to investigate how single-point variations may affect internal communication networks in SHV enzymes. We compared the structural and dynamic responses of SHV-1, SHV-2 and SHV-38 to the removal of a non-covalent active site ligand (sulbactam, Fig. S1†). Cα deviations after 5 ns of non-equilibrium simulation were compared with the equivalent time points of the unperturbed system, averaged over 200 non-equilibrium simulations (5 equilibrium simulations (Fig. S2 and S3†) with non-equilibrium simulations started from snapshots taken every 5 ns from 50 ns to 250 ns). These deviations reveal communication networks that differ between the variants (Fig. 2b, c and S4†).

The networks identified from the D-NEMD Cα deviations show connections of multiple regions to the active site in all three SHV variants (Fig. S5†). For all three enzymes, the α8–α9 loop (residues 193–200) and α10 (residues 215–230) helix and part of the Ω-loop (residues 171–180) all show significant D-NEMD Cα deviations, with moderate deviations also evident for the α3 and α4 helices (Fig. S5†). However, both SHV-2 and SHV-38 show different structural responses to the perturbation, compared to SHV-1. The average difference in deviation between SHV-1 and SHV-2 is −0.02 nm, and between SHV-1 and SHV-38 is −0.0005 nm. The response of SHV-2 is greater than that of either SHV-1 or SHV-38 in multiple regions, including the Ω-loop (residues 171–180) that borders the catalytic site (Fig. 2a, b and S4, S5†). SHV-2 therefore has more tightly correlated movements, and is more responsive to active site perturbation, than either SHV-1 or SHV-38. Our data indicate that the G238S substitution in SHV-2, that is considered to facilitate cephalosporin hydrolysis through local expansion of the active site, also exerts a more generalised effect upon enzyme dynamics *via* the intramolecular network.^51^ While SHV-1 and SHV-38 have similar response amplitudes, there are multiple significant differences at specific positions. For example, there is a large deviation at residue 175 in SHV-1, whereas the corresponding peak in SHV-38 is at residue 178. Hence, while the same regions are involved in the communication networks within each SHV enzyme, the precise architecture of each network differs between variants (Fig. 2c and S4, S5†).

The results presented here indicate that individual point mutations influence the global dynamic behaviour of the enzyme through changes to correlated motions. In turn, these changes in dynamical networks can apparently affect activity. The results indicate that the precise architecture of such networks (*i.e.* the locations of nodes showing statistically significant deviations between equilibrium and non-equilibrium simulations) reflect activity towards specific types of β-lactam substrate (*i.e.* oxyiminocephalosporins for the extended-spectrum SHV-2 enzyme and carbapenems in the case of SHV-38). Such networks provide a mechanism by which remote regions of proteins are connected to regions of direct functional interest, such as the active site, with the implication that changes within them can modulate activity.

### Predicting sites of mutation that affect enzymatic activity

The KPC-2 β-lactamase is an enzyme that efficiently hydrolyses carbapenems, the most potent β-lactam antibiotics, active against the broadest range of Gram-positive and Gram-negative bacteria of any β-lactam class.^52^ Previous D-NEMD simulations revealed a network of residues that connect an allosteric ligand binding site to the KPC-2 active site region, providing first indications that these simulations can identify distal regions that modulate enzyme activity.^23^ We use D-NEMD simulations here to identify regions significantly affected by a perturbation, in this case removal of an orthosteric ligand (heteroaryl phosphonate, compound 2,^28^ Fig. S1†); such regions potentially contain sites distant from the active site that affect activity (either turnover or substrate spectrum). To verify that the calculated networks were not simply capturing random motions of the system at equilibrium, a comparison of the networks calculated by D-NEMD simulations with a control simulation comprising a ‘null’ perturbation (where atomic velocities within the Boltzmann distribution were randomised but bound ligand was retained) was performed (Fig. S6†). Comparison of the networks calculated from the D-NEMD approach, and those calculated from the ‘null’ perturbation, provides greater stringency in discriminating significant structural responses to the perturbation from natural fluctuations.^22^

KPC-2 and SHV β-lactamases have highly similar global structures (RMSD of 1.5 Å, over 241/265 Cα atoms). However, the networks calculated after active site ligand removal differ between the two enzymes. Deviations are observed in the KPC-2 α2–β4 (residues 81–93) and α11–β7 (residues 226–236) loops, but neither region showed prominent deviations in the SHV variants (Fig. 2 and S5†). The participation of the α2–β4 loop in the dynamical networks in KPC-2 indicates strongly correlated behaviour between this distal region of the protein and the active site, despite these being relatively far apart (26.3 Å distance between the Cα atoms of the catalytic Ser70 and Gly89 in the α2–β4 loop). Previous application of the D-NEMD approach, removing an allosteric ligand from a binding site distant from the KPC-2 active site, also identified the α2–β4 loop as part of a dynamic network connecting the allosteric site to the active site.^23^ Taken together, these results indicate that this region, identified by the D-NEMD approach, is one where mutation may modulate enzymatic activity.

To test whether the D-NEMD approach could be used to predict regions in which mutations would modulate enzymatic activity, we searched the β-lactamase database^50^ to identify naturally occurring KPC variants carrying sequence polymorphisms in the α2–β4 loop. Our search identified KPC-59 as containing a single mutation within the α2–β4 loop (glycine to aspartate substitution at position 89 (G89D) compared to the parent KPC-2 enzyme); but kinetic characterizations of KPC-59 are so far unreported. Accordingly, the properties of KPC-2^G89D^ were investigated by both D-NEMD simulations and experimental kinetic and structural characterisation of the purified recombinant enzyme.

**Fig. 3:**
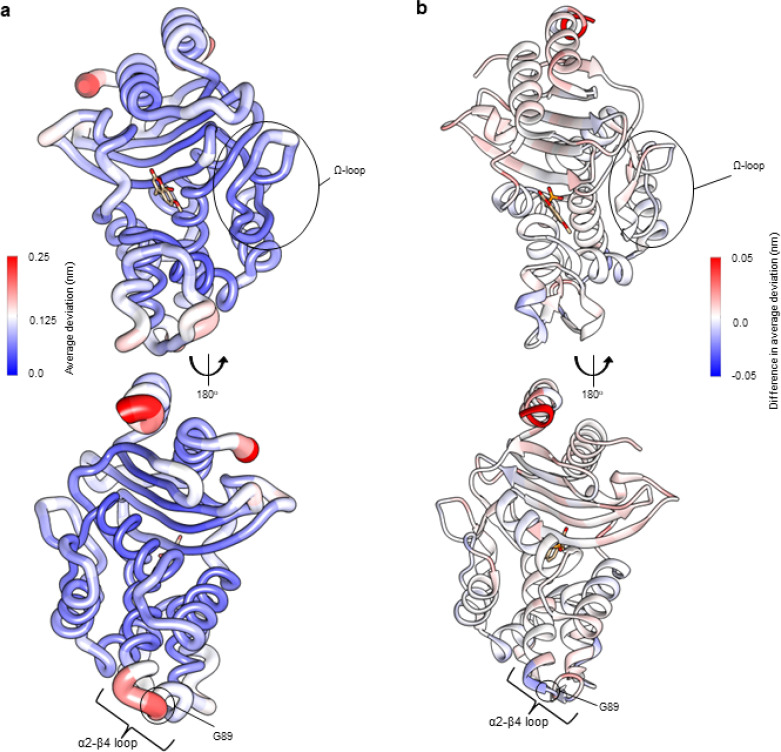
Residue deviations for KPC-2 and KPC-2^G89D^ from D-NEMD simulations. Deviations are calculated from the differences in Cα positions between perturbed and unperturbed systems (nonequilibrium *vs.* equilibrium simulations at equivalent time points) for each residue using the Kubo–Onsager relation.^16,17^ Cα deviations were then averaged over all 200 simulations (non-equilibrium simulations started from snapshots of 5 equilibrium simulations taken every 5 ns from 50 ns to 250 ns, Fig. S2 and S7†). (a) Residue deviations for KPC-2 5 ns after deletion of the active site ligand rendered onto KPC-2 crystal structure (PDB ID 6D16 (ref. 28)). (b) Difference in average residue deviations between KPC-2 and KPC-2^G89D^ 5 ns after deletion of the active site ligand rendered onto KPC-2 crystal structure (PDB ID 6D16). Positive values (red) indicate where Cα deviations were greater in KPC-2, negative values (blue) indicate where Cα deviations were greater in KPC-2^G89D^. Many difference values are statistically significant, due to the large number of replicates obtained using the D-NEMD approach (Fig. S2 and S8†). The KPC^G89D^ structure was created using the ColabFold tool^26^ (Fig. S9†). The active site ligand (compound) is shown in stick form to highlight the active site region.

A model of KPC^G89D^ was first generated using ColabFold^26^ and compound 2 positioned in the active site using ligand coordinates after superposition with the KPC-2 complex (PDB ID 6D16 (ref. 28)). For D-NEMD simulations, 200 non-equilibrium simulations of KPC-2^G89D^ were performed, in which compound 2 was removed from the active site, and the responses were compared against results for the same perturbation in KPC-2 (Fig. 3). Multiple statistically significant changes in the response amplitudes of specific regions of KPC-2 and KPC-2^G89D^ are observed (Fig. 3b and S8†): *e.g.*, at position 234 (a highly conserved residue involved in the catalytic mechanism of KPC-2 and other class A β-lactamases).^53–55^ The magnitudes of the differences in deviations between KPC-2 and KPC-2^G89D^ are smaller than those between SHV variants (Fig. 2), consistent with the greater rigidity and stability of KPC enzymes compared to other class A β-lactamases.^56^ The differences in calculated residue networks indicate that a glycine to aspartate substitution at position 89 affects the intramolecular communication network within KPC-2. This provides support for the initial D-NEMD prediction (above) that the G89D mutation affects activity.

To test this prediction, the KPC-2^G89D^ mutant was generated and purified from recombinant *E. coli*. Circular dichroism (CD) spectroscopy thermal melting experiments on the purified mutant protein indicated that the mutation does not adversely affect global stability, conferring a slight (1.1 °C) increase in melting temperature (*T*_m_) (Fig. S10†).

Strikingly, steady-state kinetic measurements (Table 1) reveal significant changes to the hydrolytic profile of KPC-2^G89D^, compared to that of wild-type KPC-2. Notably, while KPC-2 has strong carbapenemase activity, the G89D mutation results in a 100-fold decrease in catalytic efficiency (*k*_cat_/*K*_M_, Table 1) towards meropenem and a 10-fold decrease for imipenem (Fig. S1†). There is also a 10-fold decrease in the rate of turnover of the 1st generation cephalosporin cephalothin (Fig. S1†). This substantial decrease in carbapenem and cephalothin-hydrolysing activity is combined with an increase in catalytic rate (*k*_cat_, Table 1) for hydrolysis of the 3rd generation oxyiminocephalosporin cefotaxime (Fig. S1†). Moreover, *K*_M_ values for the oxyiminocephalosporins ceftazidime and cefotaxime showed significant increases compared to the parent enzyme KPC-2. In contrast, KPC-2^G89D^ is somewhat more active against penicillin. These data show the G89D mutation to affect KPC-catalysed hydrolysis of specific β-lactam substrates, rather than exerting a general effect upon enzymatic activity. This highlights the ability of the D-NEMD approach to identify residues that modulate activity (and can increase this towards some substrates), rather than those that are directly catalytic, purely structurally significant or affect global stability, for which mutation would be expected to abolish activity towards all substrates.

**Table 1:** Steady-state kinetic parameters for hydrolysis of a range of β-lactam substrates (Fig. S1) by KPC-2 and its G89D variant. Standard errors for *k*_cat_ and *K*_M_ values are shown in parentheses

β-Lactam antibiotic	β-Lactam class	*k*_cat_ (s^−1^)		*K*_M_ (μM)		*k*_cat_/*K*_M_ (s^−1^ μM^−1^)	
		KPC-2^G89D^	KPC-2	KPC-2^G89D^	KPC-2	KPC-2^G89D^	KPC-2
Ampicillin	Penicillin	160 (5.83)	82.3 (3.92)	201 (36.3)	271 (44.7)	0.79	0.3
Cephalothin	Cephalosporin	10.8 (0.32)	112 (2.78)	45.1 (3.71)	41.6 (3.41)	0.24	2.68
Ceftazidime	Cephalosporin	2.7 (0.36)	1.9 (0.12)	1340 (220)	533 (69)	2 × 10^−3^	3.5 × 10^−3^
Cefotaxime	Cephalosporin	523 (176)	75.8 (6.61)	2470 (942)	199 (29)	0.21 (0.14^a^)	0.38
Meropenem	Carbapenem	0.2 (1 × 10^−2^)	21.5 (0.12)	7.9 (1.57)	7.1 (1.01)	0.03	3.00
Imipenem	Carbapenem	2.8 (0.12)	22 (0.43)	68.9 (10.2)	72 (3.2)	0.04	0.31

a *k*_cat_/*K*_M_ value calculated from analysis of complete progress curves. This was done for cefotaxime due to the large errors resulting from a high *K*_M_ value.

To confirm the direct impact of the G89D mutation on β-lactam turnover, rather than binding, the interactions of KPC-2 and KPC-2^G89D^ with the carbapenem meropenem were investigated in pre-steady state kinetic assays under pseudo-first order conditions, monitoring tryptophan fluorescence as previously performed with OXA-48 β-lactamase.^57^ These experiments showed that the forward and reverse rate constants *k*_1_ and *k*_−1_ for meropenem binding, and the derived *K*_D_ values, (3.7 and 5.9 μM for KPC-2 and KPC-2^G89D^, Fig. S11†), are similar for the two enzymes, indicating that the G89D substitution does not affect meropenem binding. This shows that the 100-fold decrease in activity caused by this mutation is likely due to a change in the rate of the reaction on the enzyme, suggesting the G89D substitution has an impact on the active site chemistry and thus is a catalysis-modulating mutation.

Point variants of β-lactamases can also affect the efficacy of mechanism-based inhibitors, that are used clinically in combination with susceptible β-lactams,^58^ either through changes in binding interactions or changes in turnover capability.^5,59,60^ The combination of ceftazidime with the reversible, diazabicyclooctane (DBO) inhibitor avibactam is effective against most KPC-producing organisms, which generally evade the action of mechanism-based β-lactam inhibitors such as clavulanic acid. However, KPC variants are now emerging that reduce susceptibility of producer organisms to ceftazidime–avibactam. Accordingly, we investigated the *in vitro* potency of selected DBO inhibitors against KPC-2^G89D^. No significant change was observed in the IC_50_ value of avibactam (Fig. S1†) against the KPC-2^G89D^ mutant compared to the wild-type enzyme (12.2 nM *vs.* 10 nM for KPC-2).^61^ However, the G89D mutation decreases potency of inhibition by the bulkier DBO inhibitor zidebactam (Fig. S1†), with a 10-fold increase in IC_50_ value (0.7 nM *vs.* 0.06 nM for KPC-2).

**Fig. 4:**
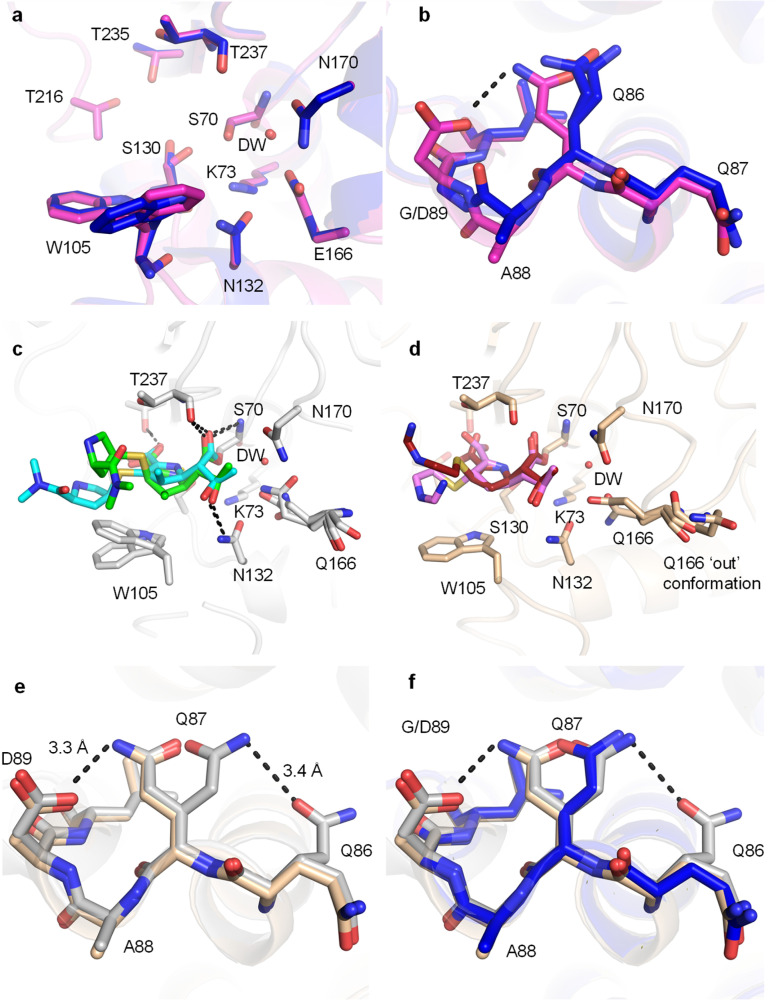
Crystal structures of the KPC-2^G89D^ mutant compared with KPC-2. (a) Active sites of uncomplexed (apo) KPC-2^G89D^ (magenta, this work) and KPC-2 (PDB ID 5UL8,^62^ blue). Residues implicated in catalysis are shown as sticks, and the deacylating water molecule (DW) as a red sphere. (b) α2–β4 loops of KPC-2^G89D^ (magenta) and KPC-2. The additional hydrogen bond between D89 and Q86 in the KPC-2^G89D^ structure is highlighted (black, dashed line). (c) Active site of the KPC-2^G89D/E166Q^ : meropenem acyl–enzyme (side chain carbon atoms in grey, Δ2 tautomer in cyan and Δ1-(2*R*) in green, Fig. S15–S17†). Hydrogen bonds between the enzyme and meropenem are shown (black, dashed lines). (d) Active site of the KPC-2^G89D/E166Q^ : imipenem acyl–enzyme (side chain carbon atoms tan, Δ1-(2*R*) tautomer in red and Δ1-(2*S*) tautomer in pink, Fig. S14–S17†). Hydrogen bonds are those observed in the KPC-2^G89D/E166Q^ : meropenem complex (distances between atoms differ, Fig. S16†). Note multiple conformations of Gln166 in both KPC-2^G89D/E166Q^ : carbapenem complexes, in particular the ‘out’ conformation of Gln166 in the KPC-2^G89D/E166Q^ : imipenem acyl–enzyme. (e) Architecture of the α2–β4 loop in KPC-2^G89D/E166Q^ : meropenem acyl–enzyme complex (grey) and the KPC-2^G89D/E166Q^ : imipenem acyl–enzyme complex (tan). (f) α2–β4 loop in the KPC-2^G89D/E166Q^ : meropenem derived acyl–enzyme complex (grey) and the KPC-2^G89D/E166Q^ : imipenem derived acyl–enzyme complex (tan) and KPC-2 : meropenem (PDB 8AKL, blue).^47^

To investigate the basis of these changes in the activity spectrum of KPC-2^G89D^, we determined X-ray crystal structures of the uncomplexed enzyme (Fig. S12 and S13†) and of its covalent complexes with the carbapenems imipenem and meropenem and with avibactam (Table S2†). The crystal structure of uncomplexed KPC-2^G89D^ revealed that, consistent with CD data, the mutation does not affect the global structure of the enzyme (Cα RMSD 0.07 Å to uncomplexed KPC-2, PDB ID 5UL8 (ref. 62)) (Fig. 4a, b and ESI Note 1†).

To capture the structures of carbapenem-derived acyl–enzyme complexes, and thus probe why the G89D substitution reduces catalytic efficiency towards carbapenems, the isosteric Glu166 to Gln166 substitution was made in KPC-2^G89D^ and the purified recombinant protein crystallised and challenged with imipenem and meropenem. The E166Q substitution renders class A β-lactamases deacylation-deficient but does not change their global structure (Fig. S13†) or prevent acylation by β-lactams.^39,47^ Diffraction data resolved meropenem- and imipenem-derived acyl–enzyme complexes with KPC-2^G89D/E166Q^ to high resolution (1.09 Å and 1.03 Å, respectively, Table S2 and Fig. S16, S17†). Carbapenem acyl–enzymes are known to tautomerise through migration of the double bond in the 5-membered pyrroline ring, making possible observation of multiple tautomers and stereomers in the same crystal structure (ESI Note 2 and Fig. S16, S17†).^47,63^ Accordingly, the KPC-2^G89D/E166Q^ meropenem acyl–enzyme was modelled in the Δ1-(2*R*) and Δ2 configurations, unlike in the meropenem derived acyl–enzyme of KPC-2^E166Q^ where only the Δ1-(2*R*) tautomer was modelled (PDB ID 8AKL).^47^ The imipenem-derived KPC-2^G89D/E166Q^ complex was modelled in both the Δ1-(2*R*) and Δ1-(2*S*) forms, as is also observed in the KPC-2^E166Q^ acyl–enzyme complex with imipenem (PDB ID 8AKK).^47^ We have previously shown that the different tautomers have different propensities to deacylate, with the initial state (Δ2) being more deacylation competent than Δ1 stereomers.^47^ There is no difference in the orientations of the ligands in either the imipenem- or meropenem-derived acyl–enzyme KPC-2^G89D/E166Q^ complexes compared to the previously deposited meropenem- and imipenem-derived KPC-2^E166Q^ acyl–enzyme complexes (PDB ID 8AKL, 8AKK,^47^ Fig. S18†) other than the additional Δ2 tautomer modelled into the meropenem derived KPC^G89D/E166Q^ acyl–enzyme. Overall, analysis of the KPC-2^G89D/E166Q^ carbapenem-derived acyl–enzyme complexes indicates that the covalent binding orientation of carbapenem substrates is not affected by the G89D mutation.

In both KPC-2^G89D/E166Q^ : carbapenem-derived acyl–enzyme structures reported here, residues (165–170) in the catalytically essential Ω-loop are highly flexible, shown *e.g.* by multiple conformations of Gln166 and high *B*-factors (Fig. S19 and S20†). The multiple conformations of Gln166 include an ‘out’ conformation (Fig. 4d and S20†) in the imipenem-derived acyl–enzyme complex, where the Gln166 side chain faces bulk solvent rather than the bound carbapenem. This ‘out’ conformation of residue 166 is adopted when KPC-2 forms acyl–enzyme complexes with substrates that deacylate poorly.^39,64^ Multiple studies of a range of class A β-lactamases show that the conformational stability of the Ω-loop in β-lactam derived acyl–enzyme complexes correlates strongly with the ability to turn over that substrate,^39,47,65,66^ suggesting that the high mobility of the Ω-loop contributes to the reduced activity of KPC-2^G89D^ towards carbapenems. *B*-Factor (adjusted, *B*′-factor) analysis of KPC-2 acyl–enzyme complexes with different β-lactam substrates from previous studies highlights a negative correlation between the stability of the α2–β4 loop (containing residue 89, Fig. 3) and the stability of the Ω-loop (Fig. S21†), *i.e.* structures with high *B*′-factors for the α2–β4 loop have low *B*′-factors for the Ω-loop.^39,47,62^ In both acyl–enzyme structures of KPC-2^G89D^ presented here, the α2–β4 loop forms extra hydrogen bonds, between residues D89 and Q87, that are not observed in equivalent KPC-2 complexes (Fig. 4e and f). These hydrogen bonds result in lower *B*′-factors for residues in the α2–β4 loop, which, combined with the observed instability/high mobility of the Ω-loop, is consistent with the observed inverse correlation between the two loops (ESI Note 2 and Fig. S21†).

A water molecule is positioned for deacylation in both crystal structures of carbapenem-derived acyl–enzyme complexes of KPC-2^G89D/E166Q^ (DW, Fig. 1 and 4c, d). This deacylating water molecule (DW) is required to resolve the covalent complex formed between antibiotics and class A β-lactamases.^6,47,67,68^ Appropriate positioning and orientation of the DW, *e.g.* through interaction with the side chains of residues 166 and 170 (Fig. 4 and S16†) promotes turnover of β-lactam acyl–enzymes.^69,70^ The high resolution of the acyl–enzyme complexes presented here allows for refinement of occupancies, including for active site water molecules. While the meropenem-derived KPC-2^G89D/E166Q^ acyl–enzyme complex contains a water molecule in the DW position, it is refined at low occupancy (0.58) compared to the structure of the parent KPC-2^E166Q^ : meropenem at similar resolution (occupancy 1.00, PDB 8AKL^47^). In contrast, in the imipenem-derived KPC-2^G89D/E166Q^ acyl–enzyme complex (a substrate for which, compared to meropenem, KPC-2^G89D^ has an increased catalytic rate (Table 1)), the DW could be refined at full occupancy (1.00) with a lower *B*-factor (Table S3†). However, the existence of multiple conformations of Glu166 indicates that stable hydrogen bonding between this residue and DW only occurs in a fraction of the imipenem derived acyl–enzyme population. Thus, increased flexibility of the Ω-loop in acyl–enzymes of KPC-2^G89D^ can reduce the stability/occupancy of DW, or reduce stable interactions with Glu166, consequently impairing deacylation.

We also determined the crystal structure of the covalent complex of KPC-2^G89D^ with the DBO inhibitor avibactam. Consistent with previous studies of DBO inhibitors, bound avibactam is observed as a mixture of the sulphated and desulphated forms (Fig. S22–S24†).^40,71^ Structural comparisons showed the orientations of bound avibactam, and its interactions with the KPC-2^G89D^ active site, to be near-identical to those observed in the wild-type structure, consistent with the minimal difference in IC_50_ values (Fig. S23†). These data highlight the selective impact of the G89D substitution on activity of KPC-2 towards substrates/inhibitors, further strengthening the conclusion that networks identified by D-NEMD contribute to specificity as well as overall catalytic activity.

## Conclusion

Here, we combine simulations and experiments to identify functionally important differences in the dynamics of β-lactamase enzymes. Simulations indicate sites of distal mutations likely to affect activity, with this prediction validated by experiment. In our initial investigations, D-NEMD simulations identify communication networks that differ between SHV variants. The extended-spectrum SHV-2 variant, with efficient activity against the broadest range of substrates, shows the greatest per-residue deviations. The precise architecture of intramolecular networks may reflect differences in the activity spectrum of the enzyme variants against specific substrates, as evidenced by differences in per-residue deviations between the SHV-1 and SHV-38 variants, which differ in activity towards carbapenems. Second, the communication networks identified by D-NEMD identify regions in β-lactamase enzymes that affect activity, in particular residues remote from the enzyme active site. Simulations of KPC-2 identified the α2–β4 loop as a participant in an allosteric network that connects distal regions of the protein to the active site. Thus, using the D-NEMD approach we predicted that mutation in this region may modulate enzymatic activity. Experimental characterisation of the (previously unstudied) KPC2^G89D^ variant substantiates this conclusion and supports the importance of intramolecular communication networks to activity:^23^ we show that this mutation selectively changes activity towards specific β-lactams, in particular reducing carbapenem hydrolysis. Our identification of the α2–β4 loop as part of a dynamic network in KPC-2, extending the findings of our previous study applying D-NEMD to study removal of an allosteric ligand from KPC-2,^23^ and our subsequent experimental validation of the prediction that mutations in this region modulate KPC-2 activity, then implies the possibility that this might present a binding site for novel inhibitors of class A β-lactamases. Of note, previous studies have reported crystal structures of class A β-lactamases with ligands bound at a site that includes the α2–β4 loop, supporting the potential of this region as a druggable site at which to target class A β-lactamases.^28,72^

The data presented here demonstrate that the D-NEMD technique can identify distal positions that affect turnover of specific β-lactams by class A β-lactamases. Such information may help to predict possible regions where point mutations, that modulate the spectrum of activity of KPC-2 and related enzymes, may occur. This significantly extends previous evidence that identified networks linking a putative allosterically binding ligand to the TEM-1 and KPC-2 active sites,^23^ indicating that these networks module catalytic activity of the respective enzymes. This knowledge should also guide antibiotic and inhibitor drug development aimed at pre-empting and overcoming resistance, potentially by targeting regions that form significant nodes in intramolecular communication networks with allosteric inhibitors. The D-NEMD approach should be a valuable tool to identify dynamic intramolecular interactions that affect protein structure and function, and is likely to find application in areas including drug discovery, rational enzyme and *de novo* protein design.

## Data availability

All raw MD simulations data (including equilibrium and non-equilibrium simulations) will be made freely available at the University of Bristol Research Data Repository (https://data.bris.ac.uk/). Analysis scripts will be made available upon request.

## Author contributions

MB, ASFO, JS and AJM conceived the experiments. MB performed all simulations and wet-lab experiments. ASFO provided supervision for the simulations and aided in data interpretation. CLT provided supervision in site directed mutagenesis, protein purification and crystal trials and contributed to data interpretation. PH contributed to data interpretation and supervision of wet lab experiments. YTAL assisted in site-directed mutagenesis, protein purification, crystal trials, and steady-state kinetics experiments as part of an undergraduate research project. BB performed the ‘null perturbation’ simulations on KPC-2. MB wrote the manuscript with input and edits from ASFO, CLT, PH, JS and AJM.

## Conflicts of interest

There are no conflicts to declare.

## Supplementary Material

SC-OLF-D4SC03295K-s001
