# Supplementary material for: Dynamical responses predict a distal site that modulates activity in an antibiotic resistance enzyme
Source: Chem Sci. 2024 Sep 30;15(41):17232–44. doi: 10.1039/d4sc03295k (PMC11443494; doi:10.1039/d4sc03295k)
Supplement: SC-015-D4SC03295K-s001 [file SC-015-D4SC03295K-s001.pdf]

Supplementary Information (SI) for Chemical Science.
This journal is © The Royal Society of Chemistry 2024

Supporting Information For:

Dynamical Responses Predict a Distal Site that Modulates Activity in an Antibiotic Resistance Enzyme

Michael Beer[1,2], Ana Sofia F. Oliveira[2], Catherine L. Tooke[1,ǂ], Philip Hinchliffe[1], Angie Tsz Yan Li[1,#], Balazs Balega[2], James Spencer[*,1], Adrian J. Mulholland[*,2]

1 School of Cellular and Molecular Medicine, University of Bristol, Bristol, United Kingdom, BS8 1TD

2 Centre for Computational Chemistry, School of Chemistry, University of Bristol, United Kingdom, BS8 1TS

[*]Corresponding Authors

SI Note 1

Crystallographic Analysis of Uncomplexed KPC-2^{G89D}

Compared to the structure of wild-type KPC-2, there are no large changes in the position or orientation of active site residues, or in the location of the deacylating water molecule (DW, Figure 1, 4a, S12), important to β-lactam hydrolysis. However, there are small differences in the α2-β4 loop, where residue 89 is located, with the carbonyl oxygen of A88 flipped by 180° (but maintaining a cis conformation) so enabling an additional hydrogen bonding interaction between the side chain amide of Q87 and a side chain carboxylate oxygen of D89 in KPC-2^{G89D} (Figure 4B, S12).

SI Note 2

Crystallographic Analysis of the KPC-2$^{G89D/E166Q}$:Carbapenem Complexes

Aside from the instability of the Ω-loop, the active sites of both KPC-2$^{G89D/E166Q}$:carbapenem complexes closely resemble those of KPC-2^{E166Q}:carbapenem complexes[42]. The oxyanion hole, formed from the backbone amides of Ser70 and Thr237 is maintained, with hydrogen bonds between each amide and the carbonyl oxygen (Figures 4c, 4d, S15) present in both KPC-2$^{G89D/E166Q}$:carbapenem structures. Furthermore, a hydrogen bond interaction between the 6α-hydroxyethyl group of both carbapenems and the side chain of Asn132 is retained. This orients the hydroxyethyl group away from the deacylating water, a factor that is known to promote carbapenemase activity of class A β-lactamases[9]. Additionally, in both KPC-2$^{G89D/E166Q}$:carbapenem structures, Ser130 is oriented within hydrogen bonding distance of the β-lactam amide (Figures 4c, 4d, S15), as is also seen in the KPC-2^{E166Q}:carbapenem complexes.

It is noted that the B'-factor of the Ω-loop in the KPC-2$^{G89D/E166Q}$:carbapenem complexes is lower than is the case in other complexes modelled with poor substrates. This is due to the multiple modelled conformations of Gln166, which prevents accurate B'-factor comparison with the KPC-2^{E166Q}:β-lactam structures. The presence of multiple modelled conformations in these structures does, however, provide another

means of identifying that the Ω-loop is flexible in the KPC-2$^{G89D/E166Q}$:carbapenem complexes, whilst the α2-β4 loop region is more stable than in the KPC-2^{E166Q}:carbapenem acyl-enzyme complex structures due to its additional electrostatic interactions.

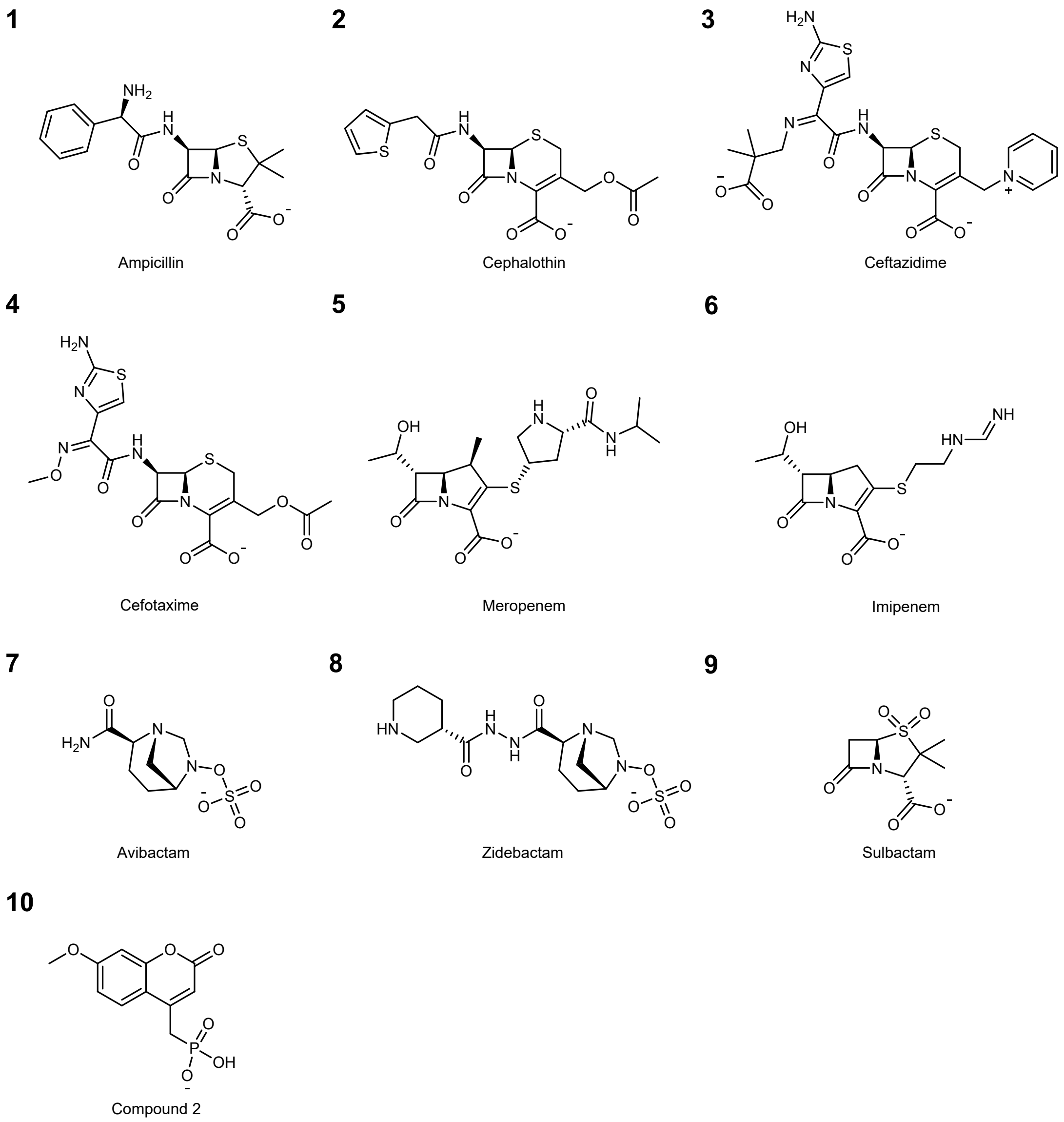

Figure S1: Structures of β-lactam antibiotics and β-lactamase inhibitors.

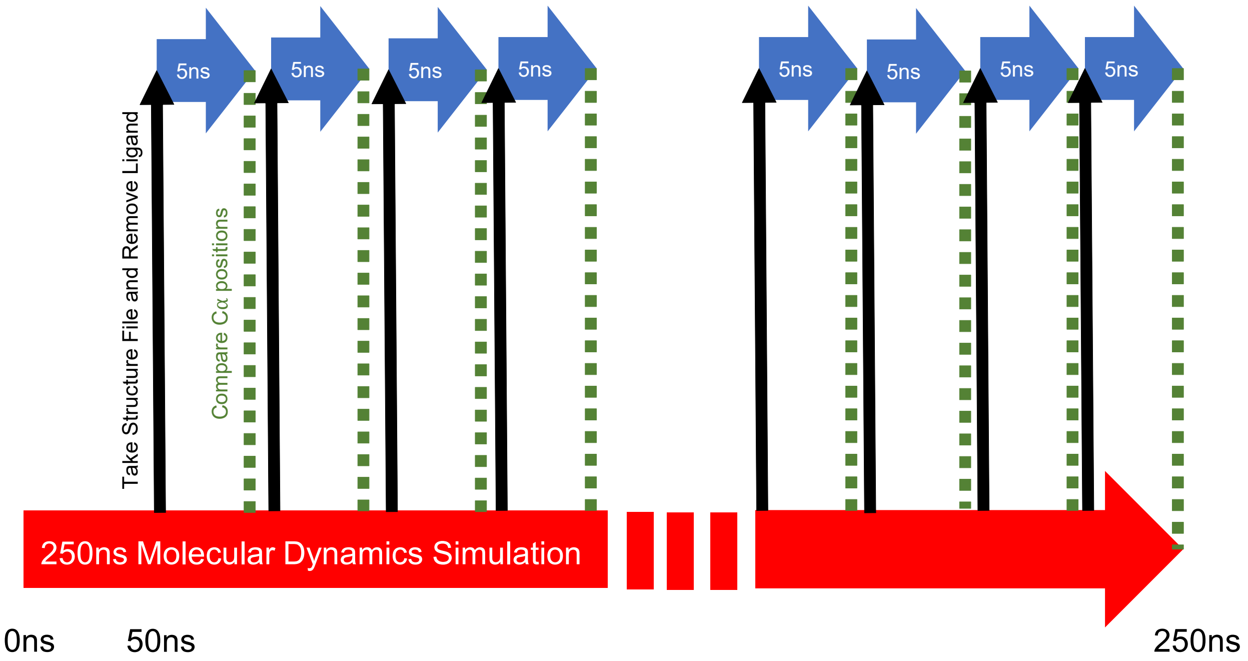

Figure S2: Theoretical framework of dynamical non-equilibrium molecular dynamics (D-NEMD) simulations. Large red arrow represents a 250 ns equilibrium molecular dynamics (MD) simulation e.g. of SHV-1 with sulbactam bound non-covalently to the active site. Black lines represent individual structure files written at each 5 ns time point on the equilibrium MD simulation from which the active site ligand atoms i.e. of sulbactam are removed. Blue arrows represent new non-equilibrium simulations starting from each of these structure files. Green dashed lines show comparisons of Cα positions between the non-equilibrium and equilibrium simulations at equivalent time points from which residue deviations are calculated [1]. This procedure is repeated for every 5ns of the 250 ns equilibrium simulation, from 50 ns onwards, i.e. totalling 40 repeats per equilibrium simulation. With 5 repeats for each system (1.25 μs of equilibrium simulation time) this results in a total 200 x 5 ns non-equilibrium simulations per system. A large number of repeats is often required to obtain statistically significant Cα deviations, in order to negate the impact of natural residue fluctuations during an MD simulation.

Table S1: Kinetic parameters for SHV-1, SHV-2 and SHV-38.

β-Lactamase	β-Lactam Antibiotic	β-Lactam Class	K_{cat} (s^{-1})	K_M (μM)	K_{cat}/K_M ($\mu M^{-1}s^{-1}$)	Vmax (μM min^{-1})
SHV-1[2]	Benzylpenicillin	Penicillin	455	20	22.8	-
	Cephalothin	Cephalosporin	10	26	0.4	-
	Ceftazidime	Cephalosporin	ND	ND	-	-
	Cefotaxime	Cephalosporin	ND	ND	-	-
	Imipenem	Carbapenem	ND	ND	-	-
SHV-2[2]	Benzylpenicillin	Penicillin	NR	3.8	NR	100
	Cephalothin	Cephalosporin	NR	NR	NR	NR
	Ceftazidime	Cephalosporin	NR	24	NR	6.5
	Cefotaxime	Cephalosporin	11	18	0.6	70
	Imipenem	Carbapenem	ND	ND	-	-
SHV-38[3]	Benzylpenicillin	Penicillin	100	13	7.7	-
	Cephalothin	Cephalosporin	5	100	0.05	-
	Ceftazidime	Cephalosporin	110	3800	0.03	-
	Cefotaxime	Cephalosporin	1	800	0.001	-
	Imipenem	Carbapenem	0.01	24	0.0004	-

ND - no detected hydrolysis.

NR – not reported.

Data obtained from Liakopoulos *et al.* [2], and Poirel *et al* [3].

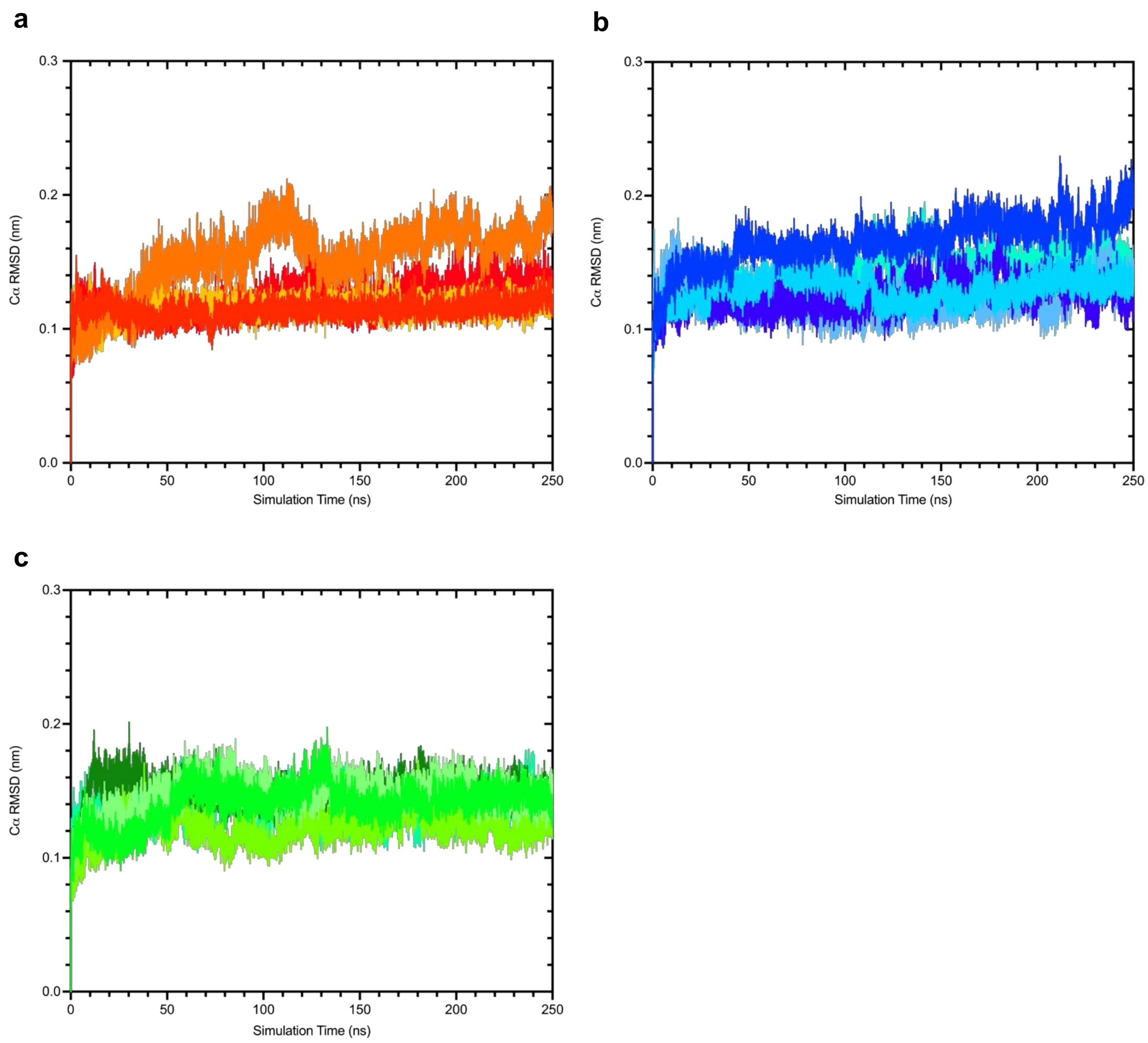

Figure S3: Temporal evolution of the all-residue average Cα RMSD for 5 repeat equilibrium MD simulations of SHV enzymes. a) SHV-1, b) SHV-2 and c) SHV-38. The Cα RMSD values were calculated relative to the starting structures. Inspection of frames with larger RMSD values indicated that large fluctuations resulted from the flexibility of the N- and C-termini.

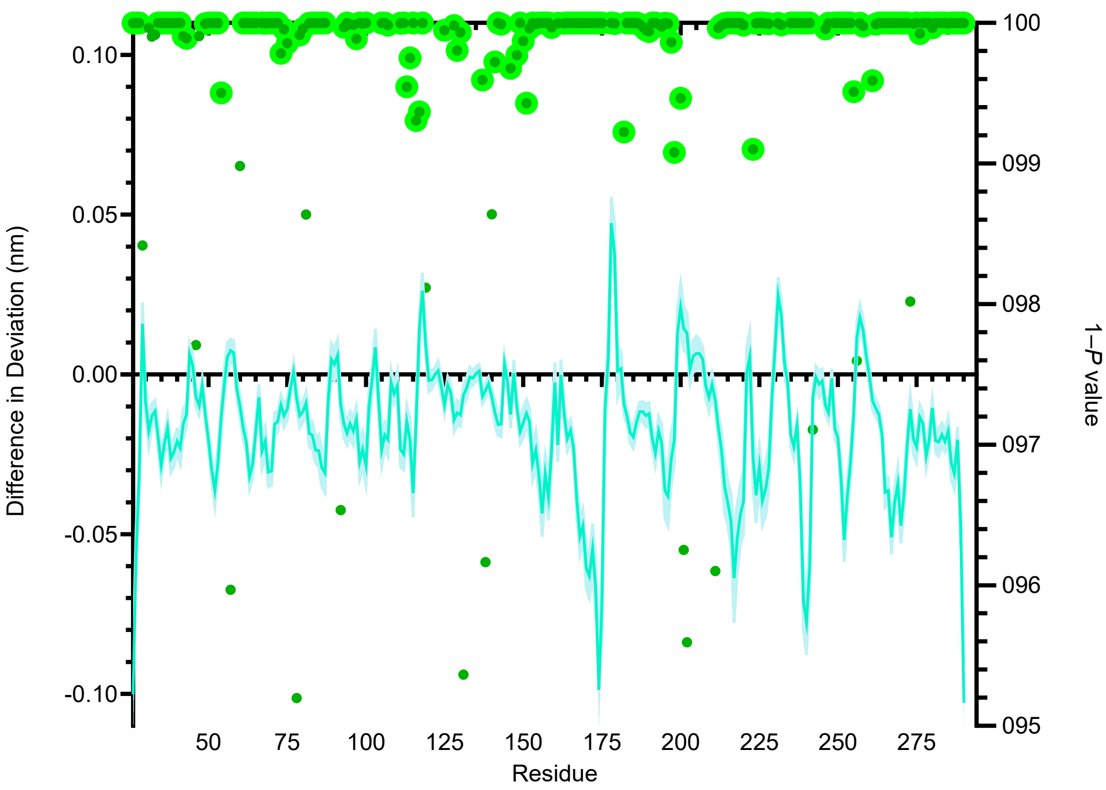

Figure S4: Differences in residue deviations for SHV-38 vs SHV-2 5ns after perturbation. Negative values indicate that a residue in SHV-2 deviated more than in SHV-38. Standard errors of the differences are shown in lighter shades above and below the difference line. Green circles denote 1 – *P* values of the difference in deviations between SHV-38 vs SHV-2 Cα deviations (right hand axis). Values with green outline are those that remain significant after false discovery rate corrections.

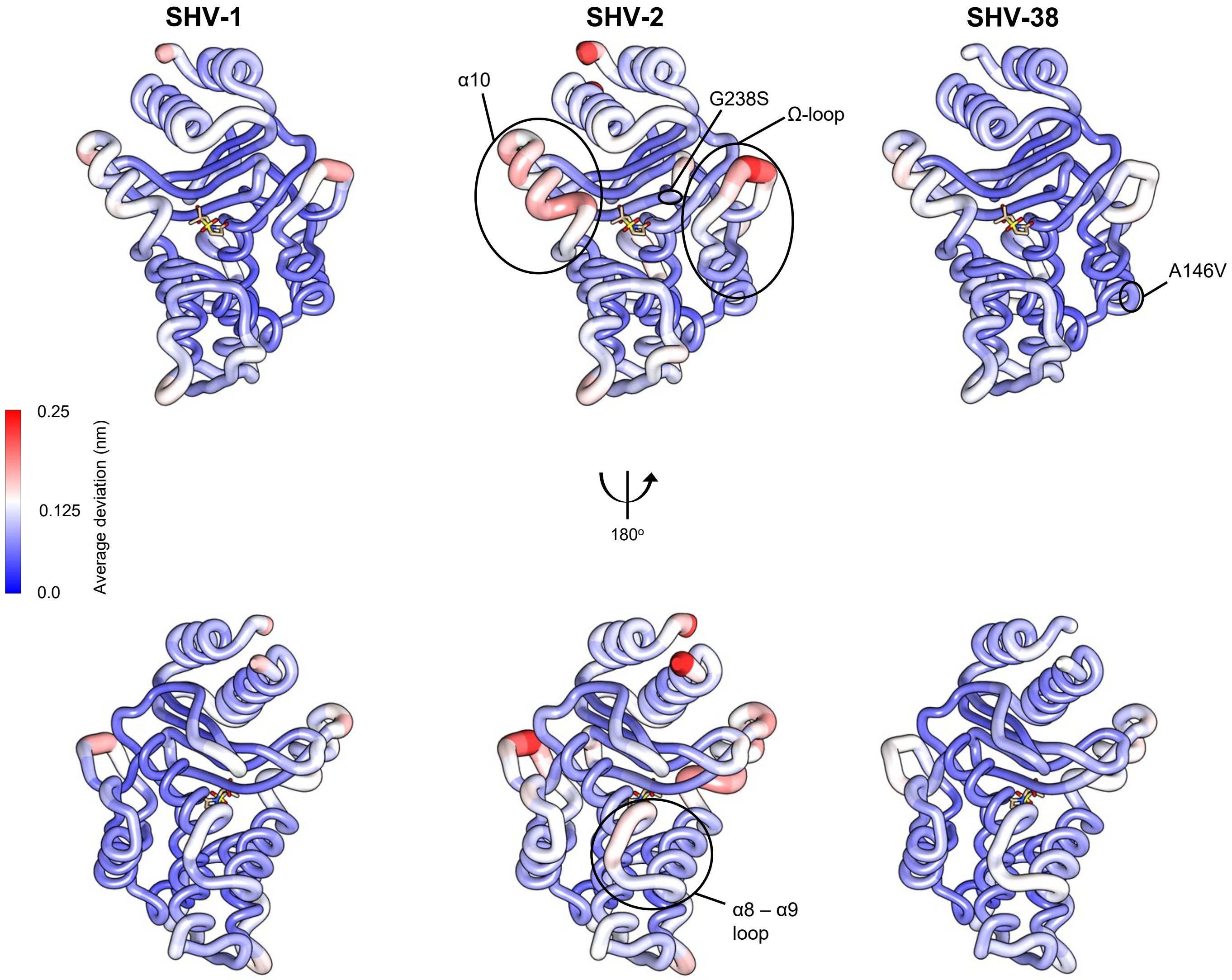

Figure S5: Average residue deviations of SHV-1, SHV-2 and SHV-38. Calculated deviations 5 ns after perturbation are rendered onto the SHV-1:sulbactam complex structure (PDB ID 4FH2 [4]). Bound sulbactam is shown as sticks to highlight the active site region. The positions of mutations in SHV-2 (G238S) and SHV-38 (A146V) and regions highlighted for their significant deviations in response to the perturbation, as described in the main text, are labelled. Images were generated using Chimera v1.17.3 [5].

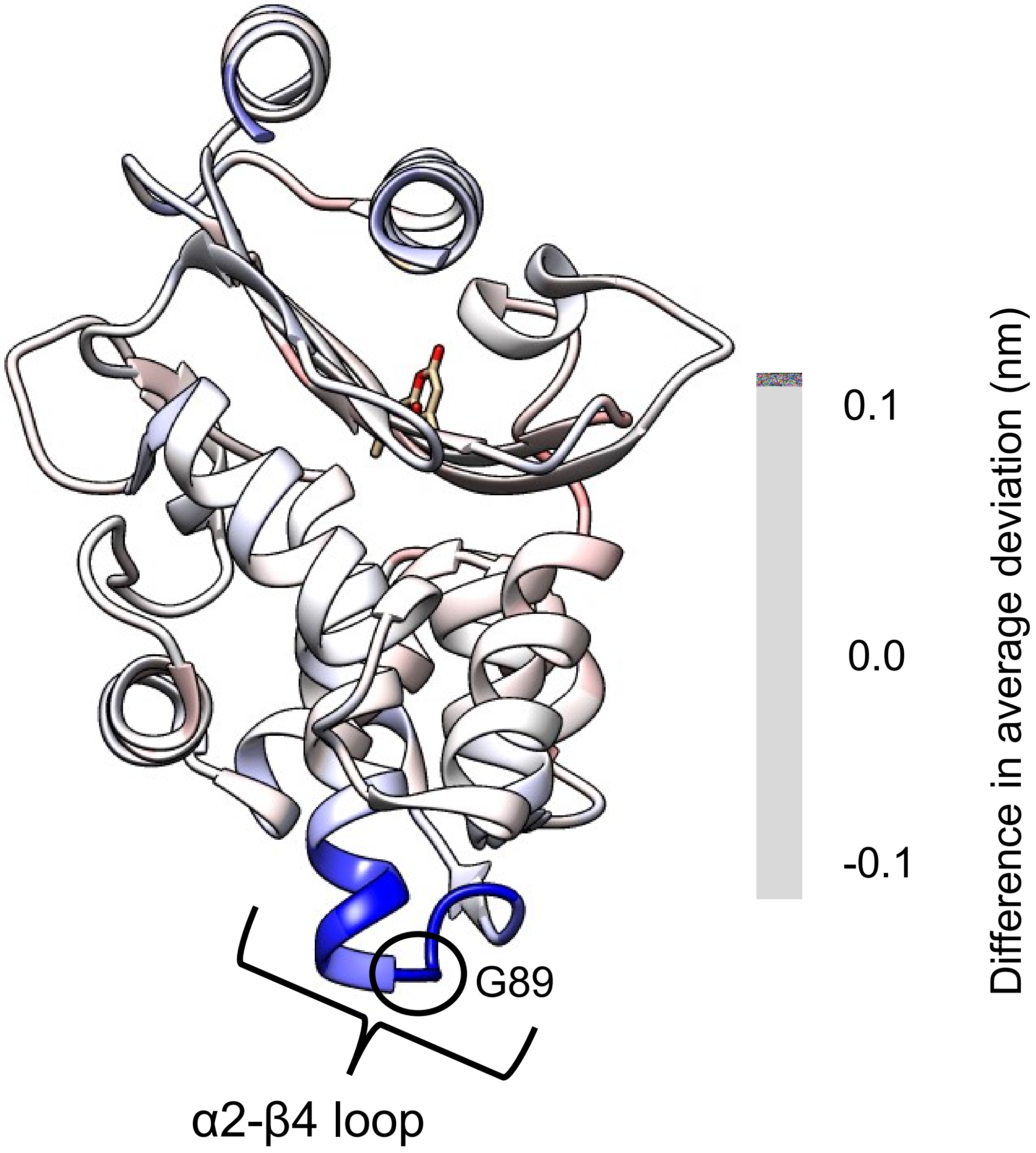

Figure S6: Structure of KPC-2 showing average differences in Cα deviation between the null perturbation and D-NEMD simulations. A value of 0 indicates no change in the Cα deviation between the null perturbation and the D-NEMD simulations, negative values indicate a reduction in Cα deviation in the D-NEMD simulations compared to the null perturbation and positive values an increase.

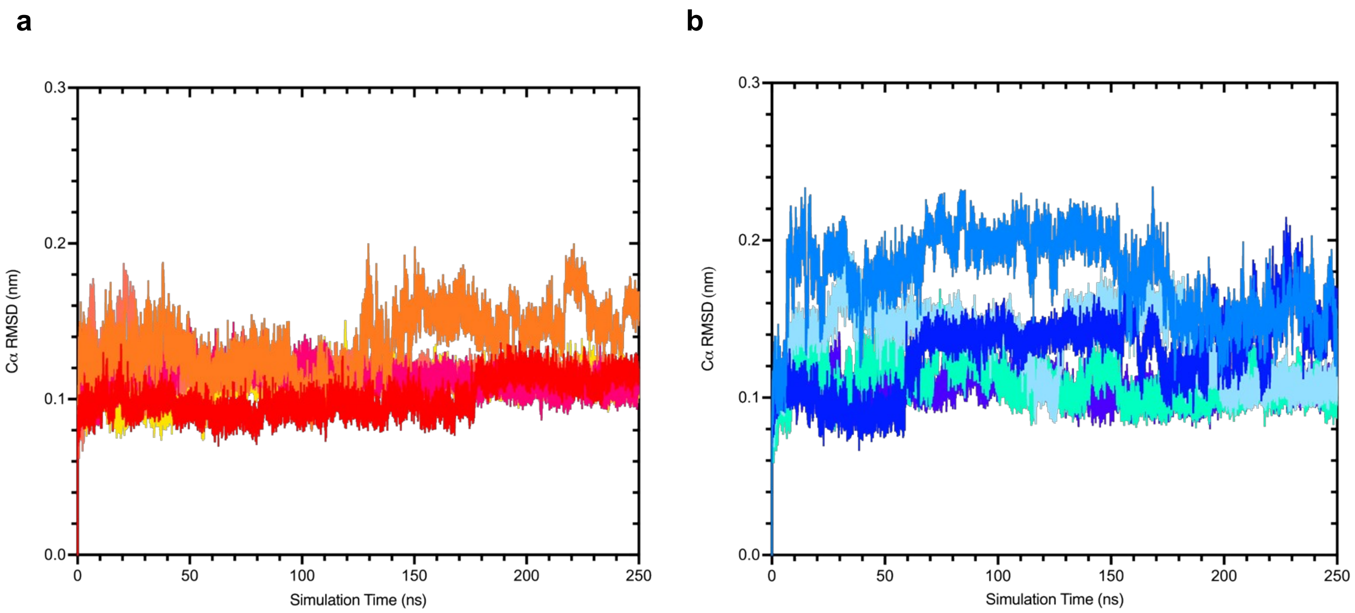

Figure S7: Temporal evolution of all-residue average Cα RMSD for all 5 repeat equilibrium MD simulations of a) KPC-2 and b) KPC-2^{G89D} enzymes. Cα RMSD values were calculated relative to the starting structures. Inspection of frames with high RMSD values indicated that large increases in RMSD reflect flexibility of the N- and C-termini.

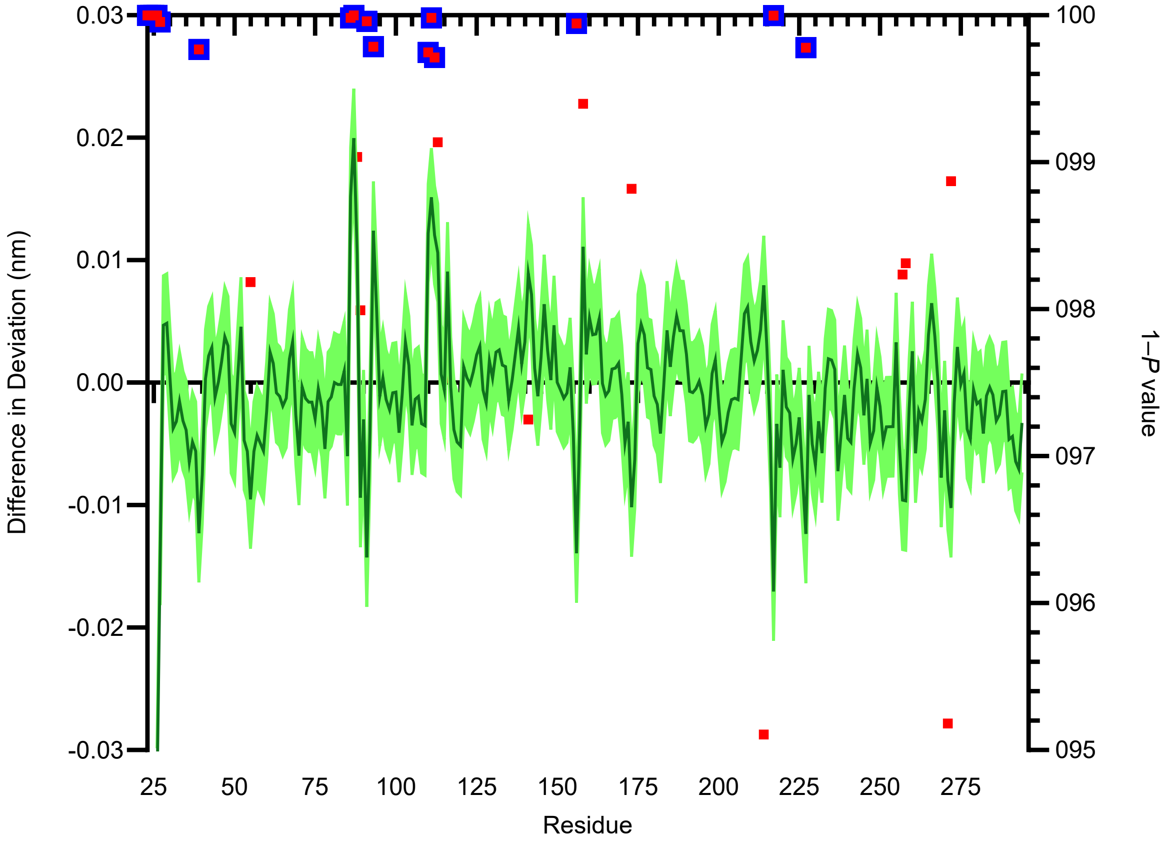

Figure S8: Differences in average Cα deviation between KPC-2 and KPC-2^{G89D}. Standard error of the difference is shown as pale green shaded region above and below the solid line. Red squares denote 1 – *P* values above 0.95 (indicating statistical significance) for the differences in average Cα deviations between KPC-2 vs. KPC-2^{G89D} (right hand axis). Boxes with a blue outline are values that remain significant after false discovery rate corrections.

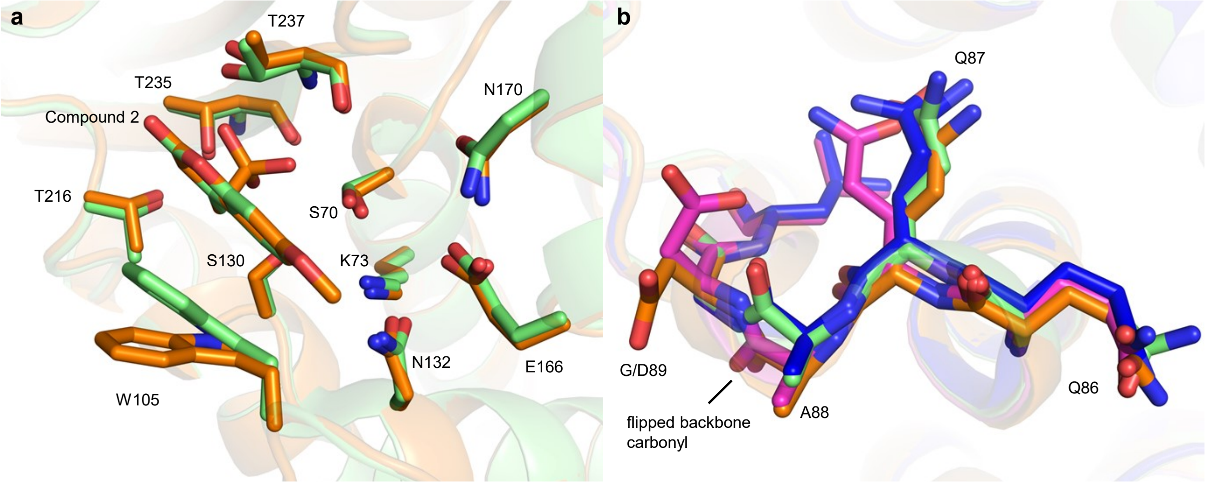

Figure S9: Validation of the ColabFold[6] KPC-2^{G89D} Model (before minimisation). a) Active site residues of KPC-2:compound 2 crystal structure (PDB ID 6D16, green) and KPC-2^{G89D} AlphaFold homology model:compound 2 structure (orange). Positions of the heteroaryl phosponate compound 2[7] are also shown. b) The α2-β4 loop (containing residue 89) loop in apo KPC-2^{G89D} (pink), KPC-2^{G89D} AlphaFold homology model:compound 2 complex (orange), KPC-2:compound 2 complex crystal structure (PDB ID 6D16, green) and uncomplexed KPC-2 (PDB ID 5UL8, blue). The AlphaFold homology model provides an accurate estimation of the structures of both the active site and α2-β4 loop and correctly captures the flipped backbone carbonyl of alanine 88.

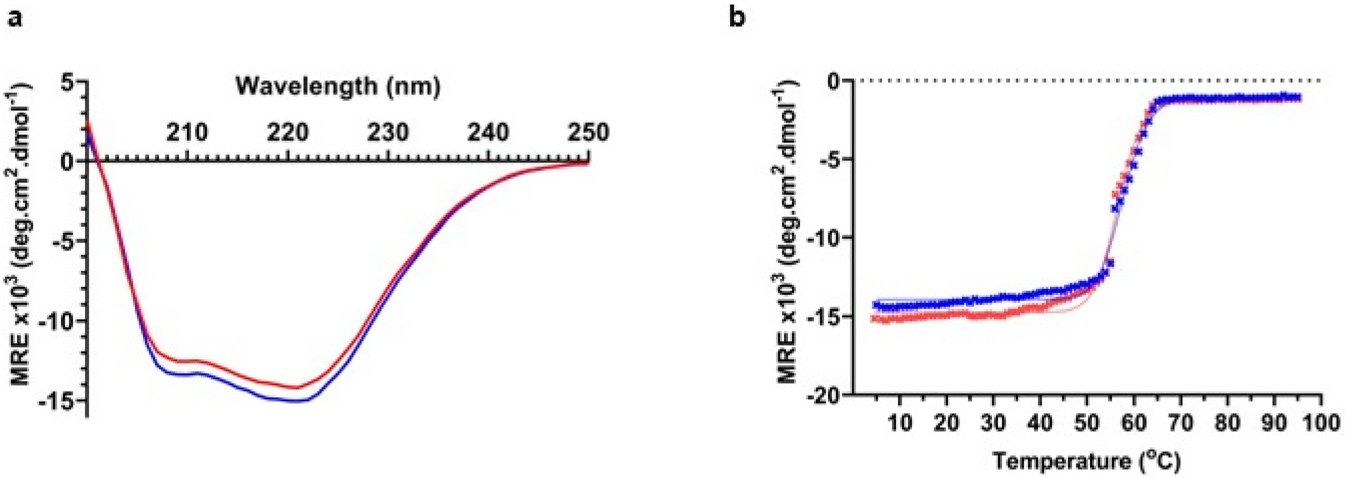

Figure S10: Circular Dichroism spectroscopy of KPC2 and KPC-2^{G89D}. a) Far-UV circular dichroism spectra of KPC-2 (red) and KPC-2^{G89D} (blue) at a protein concentration of 20 μM and at 25°C. b) Thermal melt experiment (220 nm) of KPC-2 (red, Tm 56.6°C) and KPC-2^{G89D} (blue, Tm 57.7°C). The solid line represents the non-linear regression model used to calculate Tm.

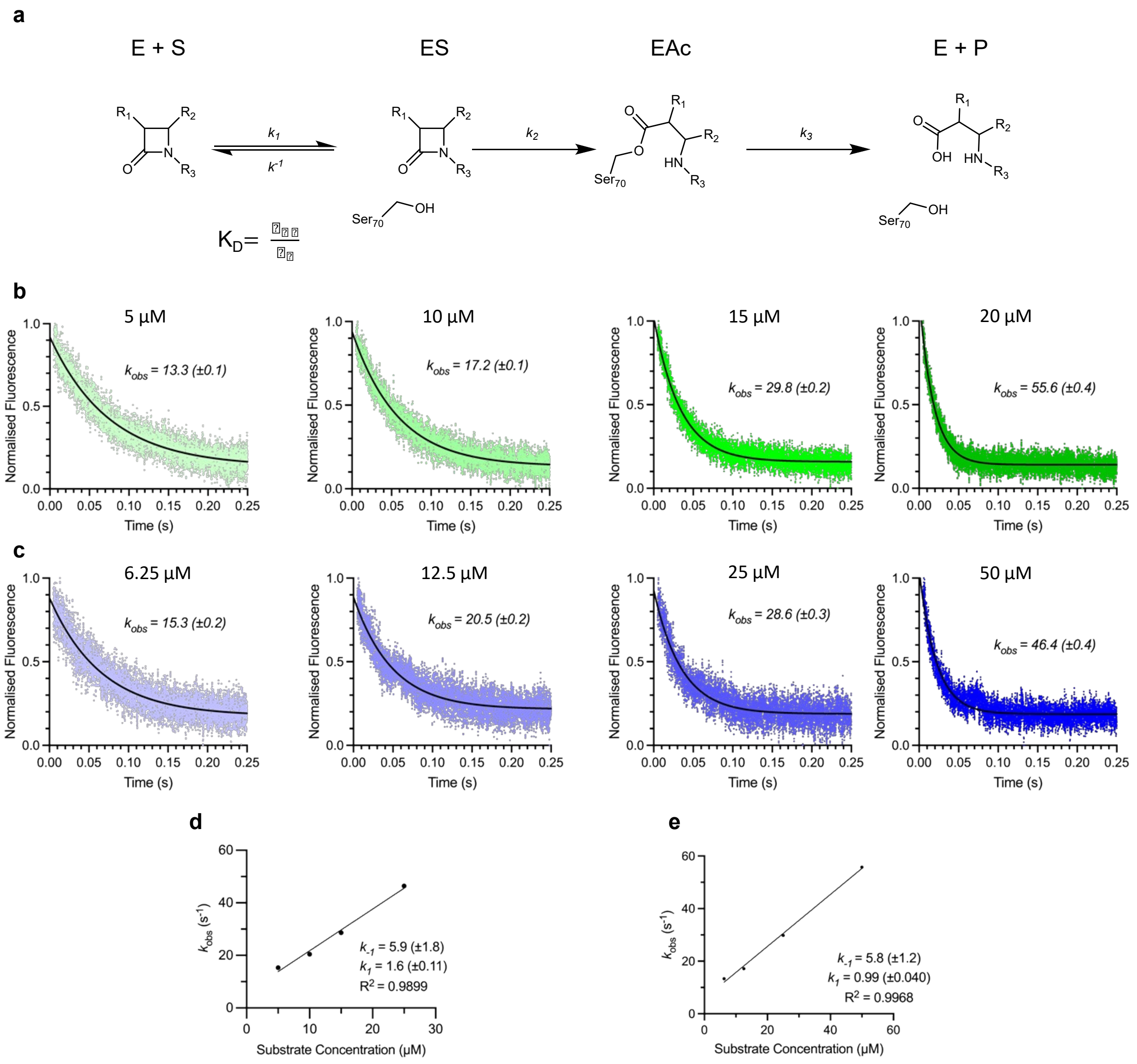

Figure S11: Binding kinetics of meropenem calculated using pre-steady state kinetics. a) Kinetic model for binding and hydrolysis of β-lactam substrates by serine β-lactamases. K_D is calculated by k_{-1} / k_1. Normalised tryptophan fluorescence curves, averaged over 5 transients, measured from 0.005 s to 0.25 s, for meropenem binding to KPC-2 (b) and KPC-2^{G89D} (both at 10 nM) (c) were used to calculate k_{obs} over a range of concentrations using one phase decay curve fitting. k_{obs} was plotted for KPC-2 (d) and KPC-2^{G89D} (e), and a linear regression used to calculate the y-intercept (k_{-1}) and the slope (k_1). The calculated K_D values are 3.7 µM and 5.9 µM for KPC-2 and KPC-2^{G89D}.

Table S2: Crystallographic data collection and refinement statistics.

	$KPC\text{-}2^{G89D}$	$KPC\text{-}2^{G89D}$:avibactam	$KPC\text{-}2^{G89D/E166Q}$	KPC-<br>$2^{G89D/E166Q}$:imipenem	KPC-<br>$2^{G89D/E166Q}$:meropenem
**PDB Code**	8RWO	8RWP	8RWQ	8RWR	8RWS
**Data Collection**					
Beamline	Diamond Light Source I03	Diamond Light Source I03	Diamond Light Source I03	Diamond Light Source I03	Diamond Light Source I03
Wavelength	0.81531	0.81531	0.81531	0.81531	0.81531
Resolution Range	59.94-1.13	45.39-1.19	45.56-1.05	45.60-1.03	36.39-1.09
Space group	P 21 21 2	P 21 21 2	P 21 21 2	P 21 21 2	P 21 21 2
Molecules/ASU	1	1	1	1	1
Cell dimensions					
a, b, c (Å)	59.94 78.62 55.89	60.14 78.49 55.64	60.36 78.66 55.89	60.21 79.45 55.68	60.12 78.75 56.16
α, β, γ (°)	90.00, 90.00, 90.00	90.00 90.00 90.00	90.00 90.00 90.00	90.00 90.00 90.00	90.00 90.00 90.00
Multiplicity	13.2 (12.9)	13.5 (13.9)	13.4 (13.3)	13.6 (13.8)	13.4 (13.2)
Completeness (%)	100.0 (100.0)	100.0 (100.0)	100.0 (99.9)	98.5 (96.1)	99.4 (97.4)
I/σ(I)	5.8 (0.5)	10.0 (0.4)	8.8 (0.3)	9.6 (0.3)	7.0 (0.3)
R_{pim}	0.063 (1.259)	0.033 (1.005)	0.036 (1.003)	0.031 (1.104)	0.042 (1.105)
$CC_{1/2}$	0.999 (0.347)	1.000 (0.295)	0.999 (0.320)	1.000 (0.347)	0.999 (0.324)
**Refinement**					
Resolution	55.89-1.13	45.39-1.19	45.56-1.05	39.73-1.03	36.39-1.09
No. reflections	99264	84567	124269	129928	110309
R-work/R-free	0.1613 / 0.1899	0.1554 / 0.1794	0.1486 / 0.1688	0.1587 / 0.1745	0.1740 / 0.1996
No. non-H atoms					
Protein	2157	2142	2087	2185	2200
Solvent	393	382	399	303	306
Ligand	-	28	-	40	52
Average B-factors					

Protein	15.6	20.5	16.3	17.1	19.9
Solvent	32.7	38.6	36.3	35.1	34.8
Ligand	-	20.9	-	19.4	25.3
R.m.s deviations					
Bond lengths (Å)	0.007	0.008	0.006	0.007	0.007
Bond angles (°)	0.966	0.999	0.907	1.175	1.019
Ramachandran (%)					
Outliers	0.00	0.00	0.00	0.00	0.00
Favoured	98.88	98.88	98.88	98.88	98.49

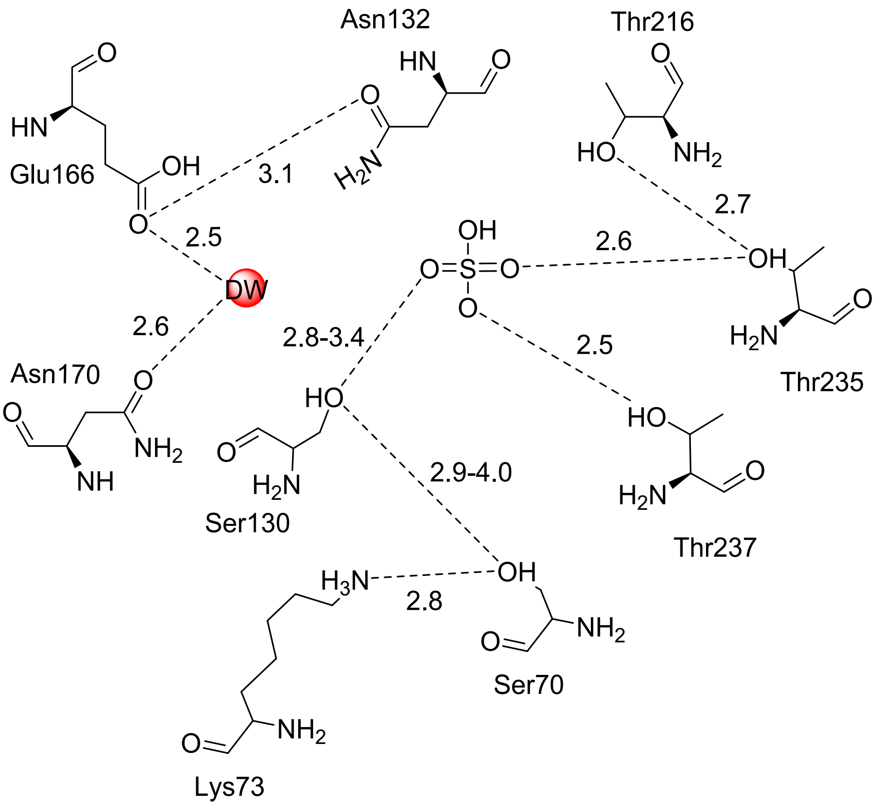

Figure S12: Active Site Interaction Diagram of uncomplexed KPC-2^{G89D}. Hydrogen bonding interactions are highlighted (black dashed line) with interatomic distances shown in Å. Distance ranges are given for hydrogen bonding around Ser130 as the Ser130 side chain was modelled in two conformations.

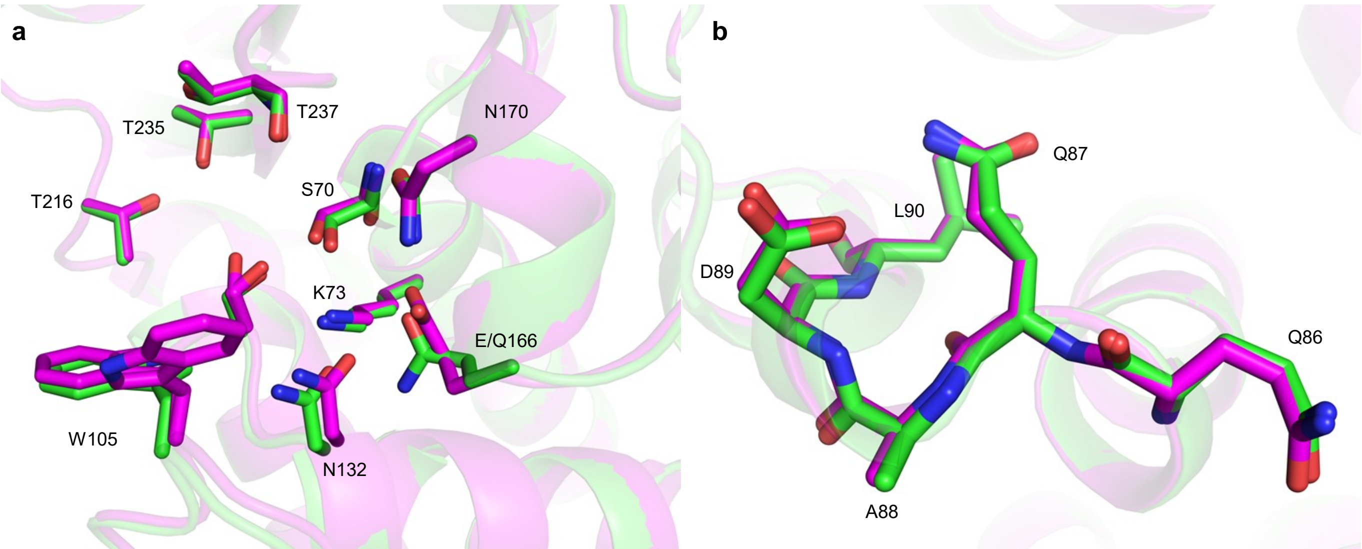

Figure S13: Structure of Uncomplexed KPC-2$^{G89D/E166Q}$. a) Active site residues of KPC-2^{G89D} (pink) and KPC-2$^{G89D/E166Q}$. b) Alignment of the α2-β4 loop in KPC-2^{G89D} and KPC-2$^{G89D/E166Q}$ structures.

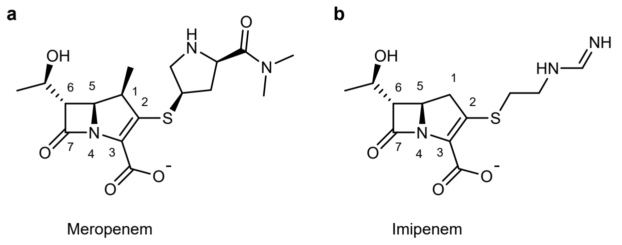

Figure S14: Atom numbering of meropenem and imipenem. The β-lactam carbonyl carbon (C-7) is the position of nucleophilic attack by Ser70, breaking the scissile β-lactam amide bond and forming the acyl-enzyme complex. Meropenem (a) and imipenem (b) differ in their C2 substituents and the presence of the meropenem 1β-methyl group (cf. imipenem 1β-hydrogen).

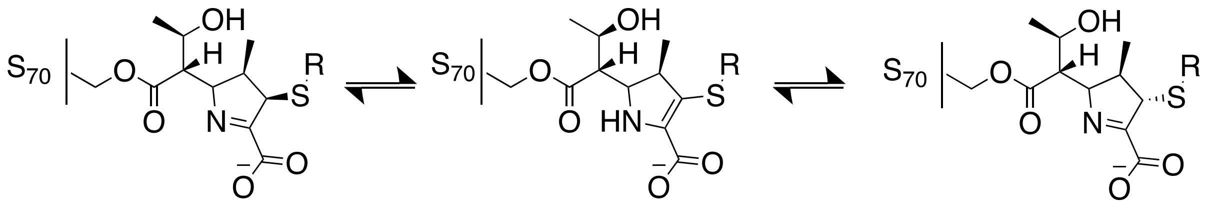

Δ1-2*R* **Δ2** **Δ1-2*S***

Figure S15. Carbapenem acylenzyme tautomerisation. In the carbapenem acyl-enzyme complex, the double bond in the pyrroline ring can migrate from C3 = C2 (Δ2, enamine) to N4 = C3 (Δ1, imine, in 2*R*- and 2*S*- configurations), resulting in three possible forms, Δ2, Δ1-(2*R*) and Δ1-(2*S*).

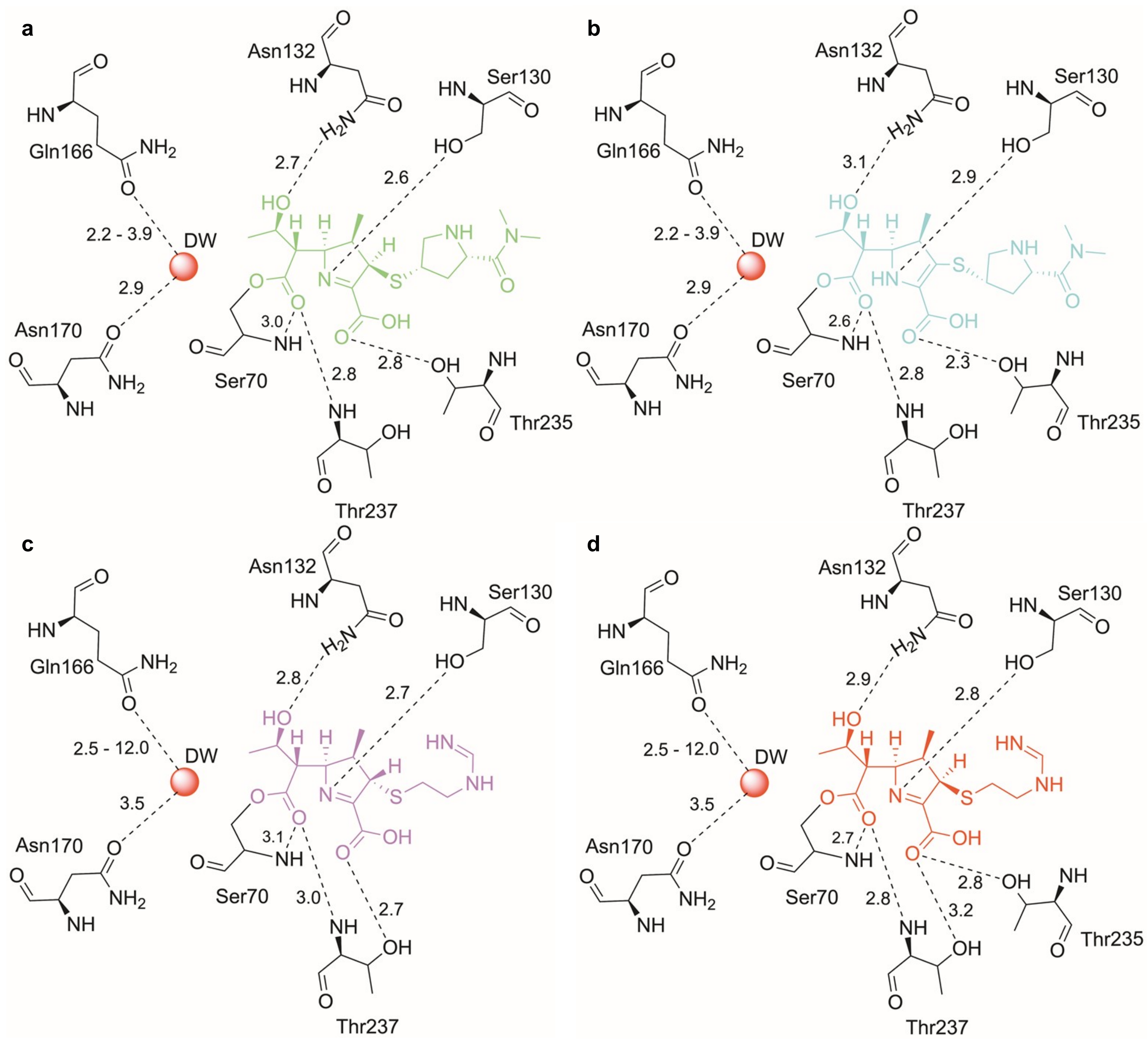

Figure S16. Ligand interactions of KPC-2^{G89D}:carbapenem complexes. a) KPC-2$^{G89D/E166Q}$:meropenem Δ1-(2*R*) tautomer. b) KPC-2$^{G89D/E166Q}$:meropenem Δ2 tautomer. c) KPC-2$^{G89D/E166Q}$:imipenem Δ1-(2*S*) tautomer. d) KPC-2$^{G89D/E166Q}$:imipenem Δ1-(2*R*) tautomer. Ranges of values for the Gln166-OE1 - deacylating water (DW) indicate minimum and maximum distances observed for multiple conformations modelled in the crystal structures. The larger distance range for imipenem reflects the modelling of Gln166 in the 'out' conformation in some conformers of the imipenem-derived structure; i.e. with the side chain pointing into bulk solvent rather than towards DW. Only 'in' conformers of Gln166 were modelled in the meropenem-derived structures. Structures were generated using LigPlot+ v2.2 [8].

a b

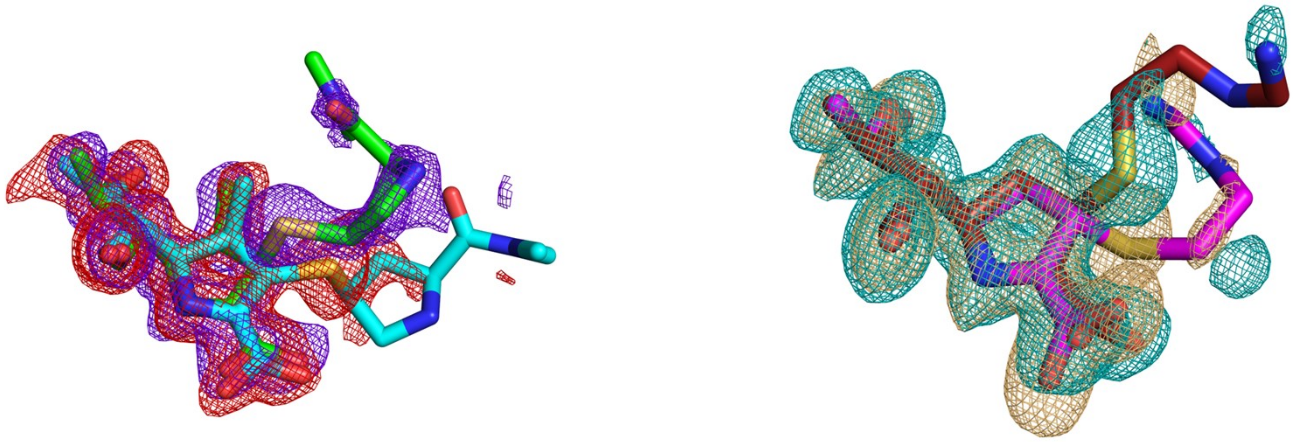

Figure S17: Omit maps of KPC-2G89D/E166Q:carbapenem complexes. Modelled ligands with *F*o-*F*c omit density, calculated after ligand removal, contoured at 3σ. a) Δ1-(2*R*) (ligand carbon atoms green, blue density) and Δ2 (ligand carbons cyan, red density) tautomers of the meropenem-derived acylenzyme. Tautomers were modelled in 54 s% and 46 % occupancies for the Δ2 and Δ1-(2*R*) tautomers, respectively. b) Δ1-(2*R*) (red ligand carbons red, cyan density) and Δ1-(2*S*) (magenta ligand, orange density) tautomers of the imipenem-derived acylenzyme. C2 substituents are known to be flexible and make few/no direct electrostatic contacts with enzyme residues, reflected in the poor electron density resolution after the exocyclic sulphur atom. Tautomers were modelled in 56 % and 44 % occupancies for the Δ1-(2*S*) and Δ1-(2*R*) tautomers, respectively.

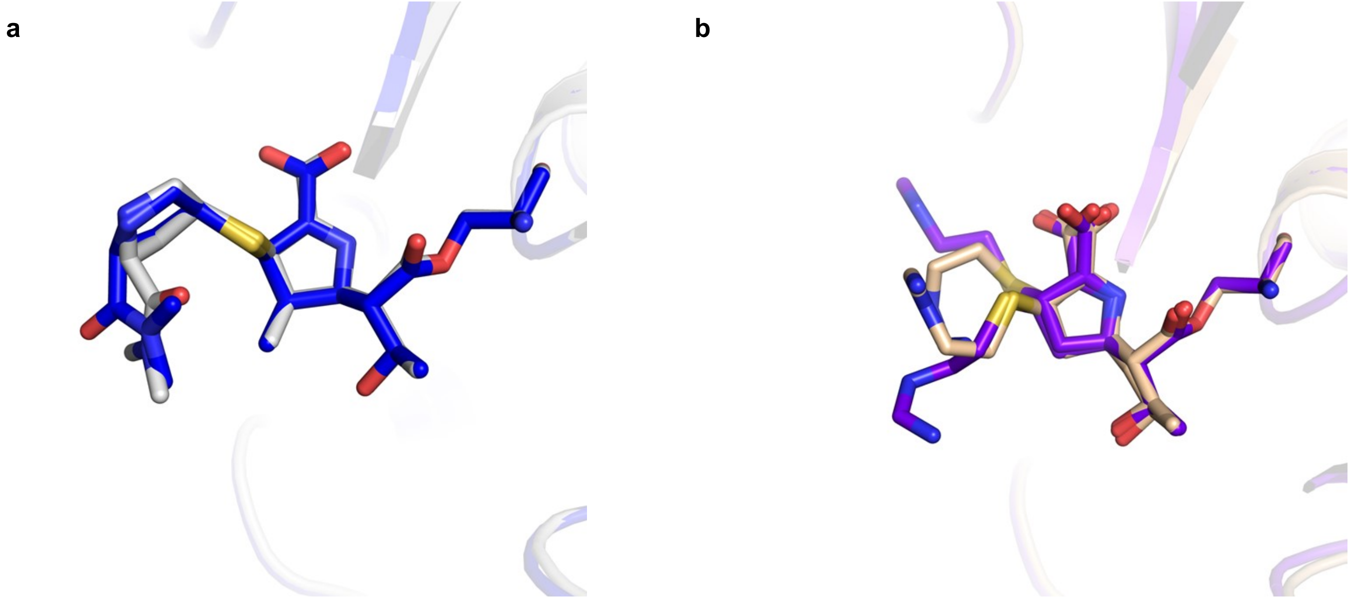

Figure S18: Carbapenem orientation in the KPC-2$^{G89D/E166Q}$ active sites. Structural alignment between (a) KPC-2$^{G89D/E166Q}$:meropenem (grey ligand) and KPC-2^{E166Q}:meropenem (PDB 8AKL, blue ligand) and (b) between KPC-2$^{G89D/E166Q}$:imipenem (tan ligand) and KPC-2^{E166Q}:imipenem (PDB 8AKK, purple ligand). The C2 substituent (Figure S14) is known to make few/no interactions with enzyme residues and consequently is highly flexible, particularly after the sulphur atom. This accounts for the difference in binding conformation in this region of the carbapenem, particularly for the imipenem molecule (b).

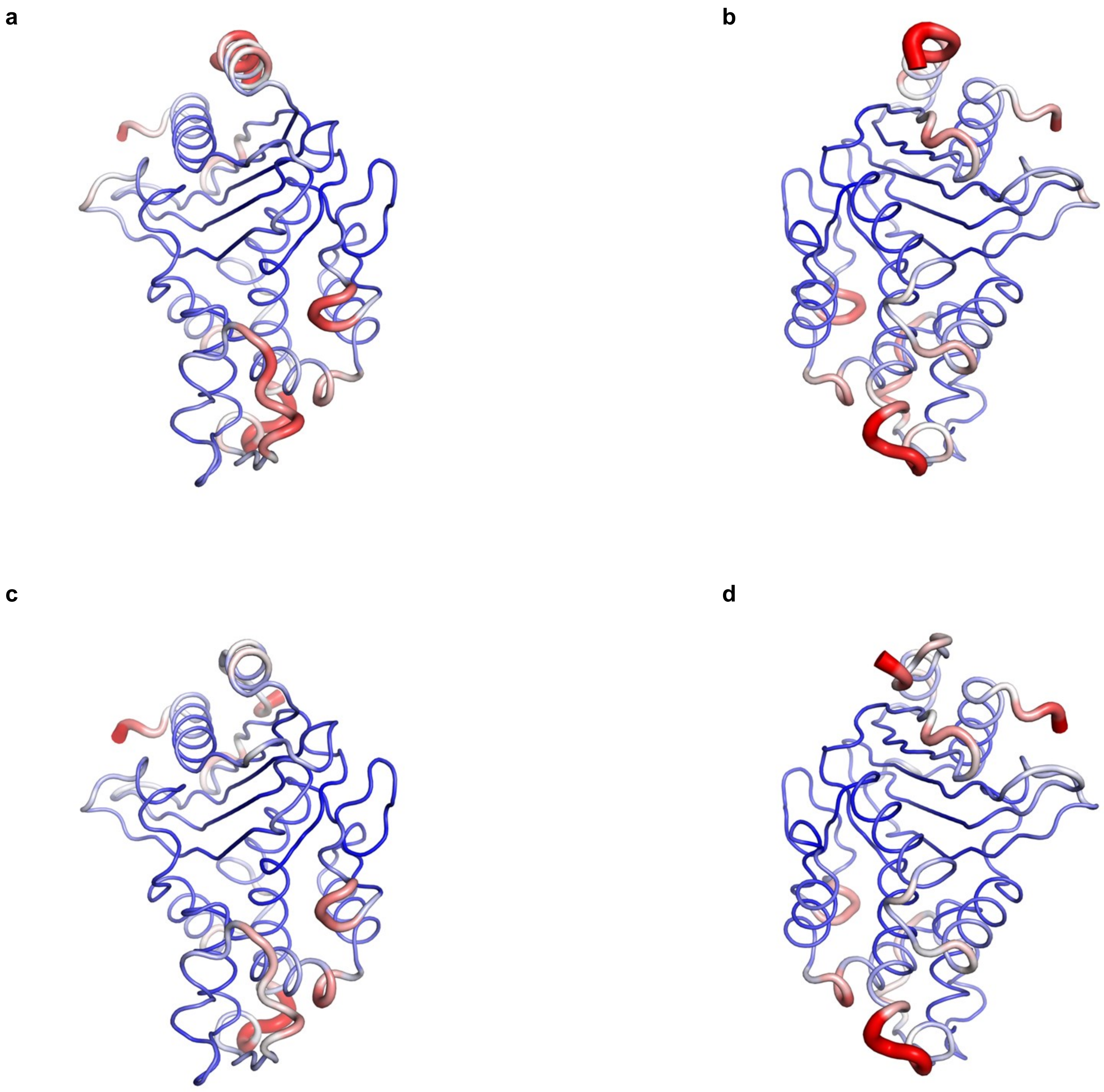

Figure S19: B-factor analysis of the KPC-2$^{G89D/E166Q}$:carbapenem complexes. B-factors rendered onto KPC-2$^{G89D/E166Q}$:meropenem (a-b) and KPC-2$^{G89D/E166Q}$:imipenem (c-d) shows high B-factors for both the Ω- and α4-β2 loops. The scale represents B-factor values between 10 (thin, blue) to 35 (thick, red).

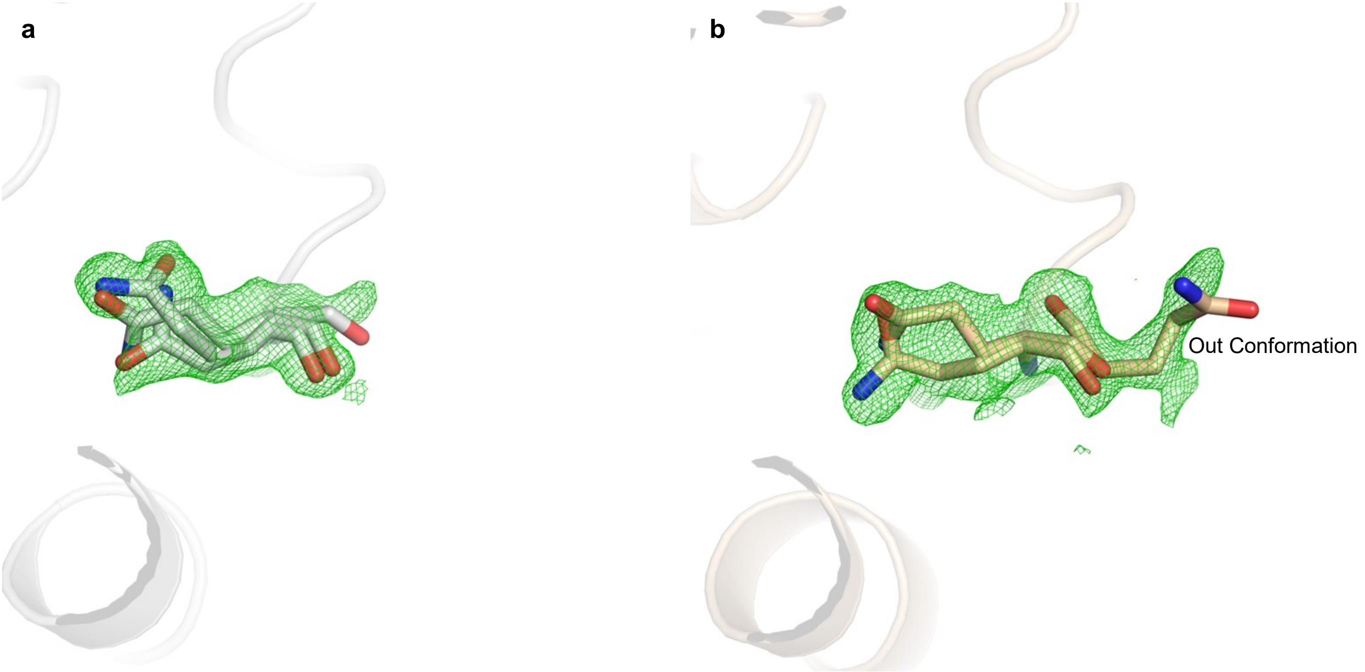

Figure S20: Polder map of Gln166 in KPC-2$^{G89D/E166Q}$:carbapenem complexes. F_o-F_c polder maps contoured at 3σ. a) Gln166 in the KPC-2$^{G89D/E166Q}$:meropenem complex. b) Gln166 in the KPC-2$^{G89D/E166Q}$:imipenem complex, showing the 'out' conformation.

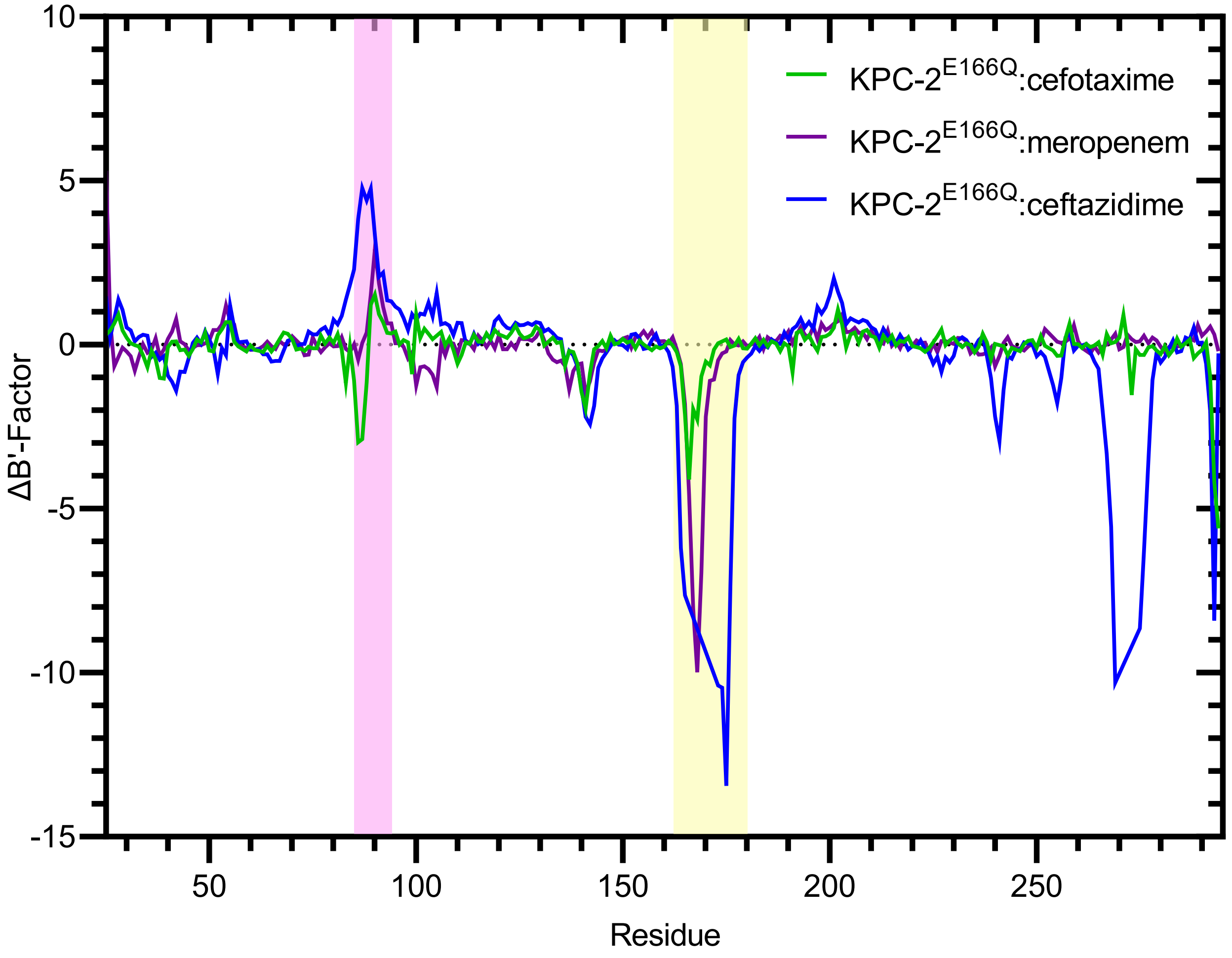

Figure S21: Difference in adjusted B-factors (B'-factors) between uncomplexed KPC-2 and KPC-2^{E166Q}:β-lactam acyl-enzyme complexes. Plot shows differences in B'-factors between uncomplexed KPC-2 (PDB 5UL8) and: KPC-2^{E166Q}:cefotaxime (PDB ID 6Z23[9], green); KPC-2 ^{E166Q}:meropenem (PDB 8AK1[10], purple) and KPC-2^{E166Q}:ceftazidime (PDB ID 6Z24[9], blue). Negative values indicate residues for which the B'-factor of the respective KPC-2^{E166Q}:β-lactam complex is greater than that of uncomplexed KPC-2. Turnover is slowest for ceftazidime (k_{cat} = 1.9 s^{-1}) and fastest for cefotaxime (k_{cat} = 76 s^{-1}), demonstrating that low B'-factors for the α2 - β4 loop (pink highlight) and high B'-factors for Ω-loop (yellow highlight) in the respective acylenzymes correlate well with turnover of specific substrates by KPC-2.

Table S3: DW occupancy and B-factor in KPC-2$^{G89D/E166Q}$:carbapenem complexes.

Structure	DW Occupancy	DW B-factor
KPC-2$^{G89D/E166Q}$:meropenem	0.58	24.49
KPC-2$^{G89D/E166Q}$:imipenem	1.00	19.74

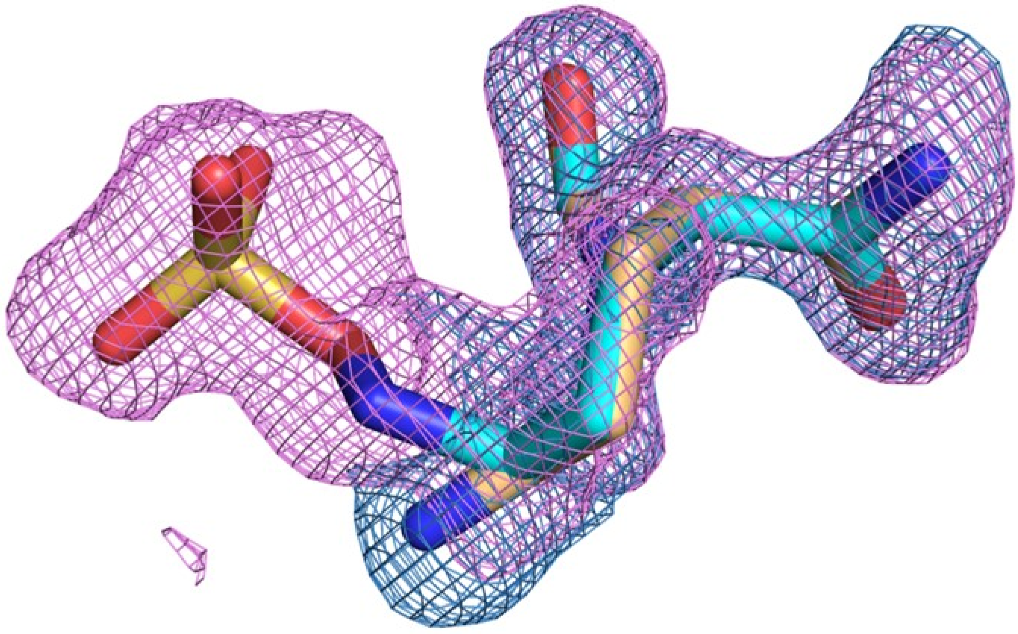

Figure S22: **Omit map density of KPC-2^{G89D}:avibactam complex.** Omit map density contoured at 3σ for sulphated (magenta mesh) and desulphated (teal mesh) avibactam structures modelled.

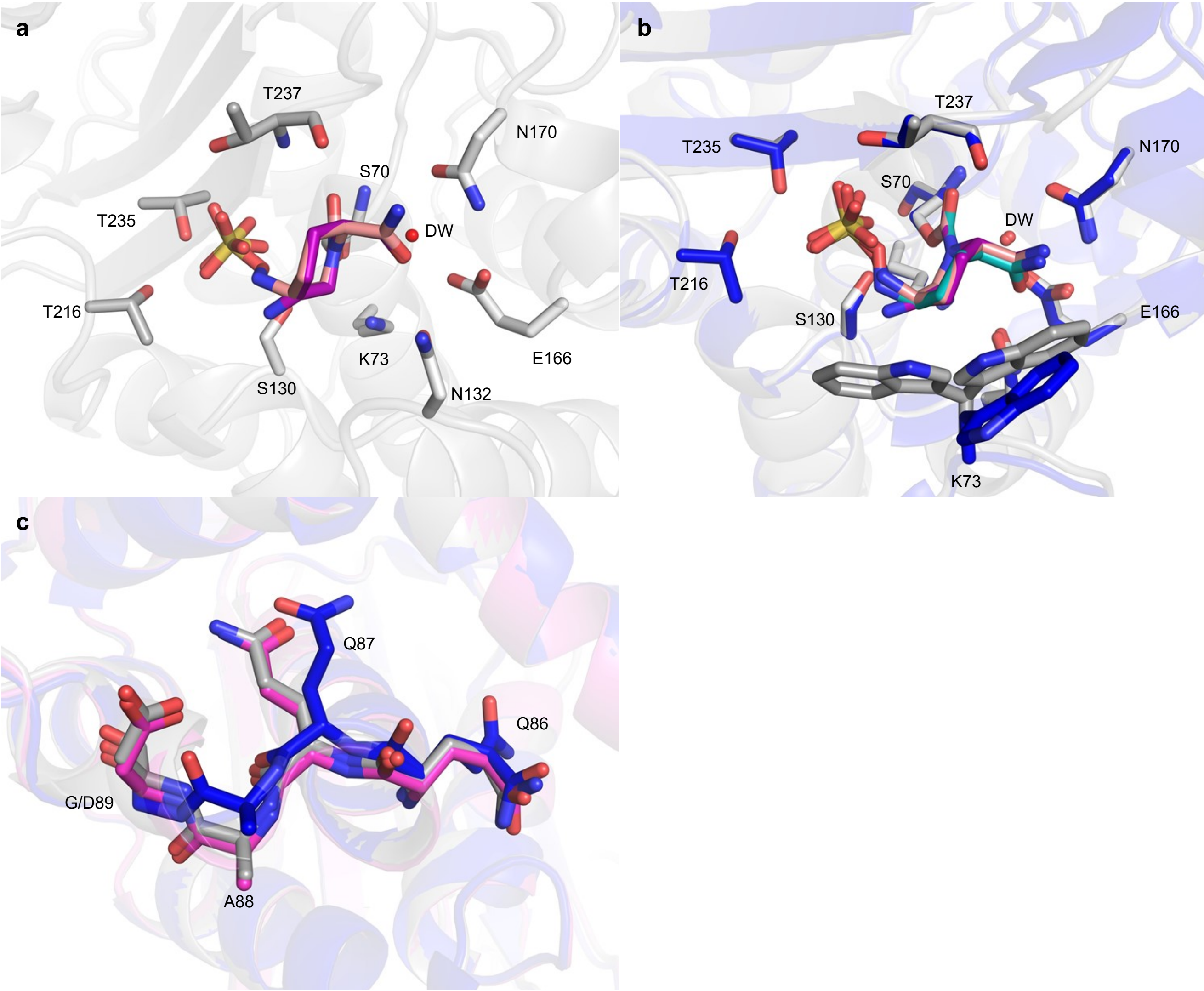

Figure S23: KPC-2^{G89D}:avibactam structure. a) Active site of KPC-2^{G89D} in complex with avibactam. Both the sulphated (orange) and desulphated (purple) forms of avibactam could be modelled. Both the sulphated and desulphated forms were refined to an occupancy of 0.5 each. b) KPC-2^{G89D}:avibactam complex structure (KPC-2^{G89D} side chain carbon atoms grey, ligands purple and orange) aligned with KPC-2:avibactam (carbon atoms blue, ligand cyan, PDB ID 4ZBE [11]). Overall RMSD between the two complexes is 0.2 Å. c) Alignment of KPC-2^{G89D}:avibactam (grey), KPC-2:avibactam (blue) and uncomplexed KPC-2^{G89D} (pink), showing the α2-β4 loop.

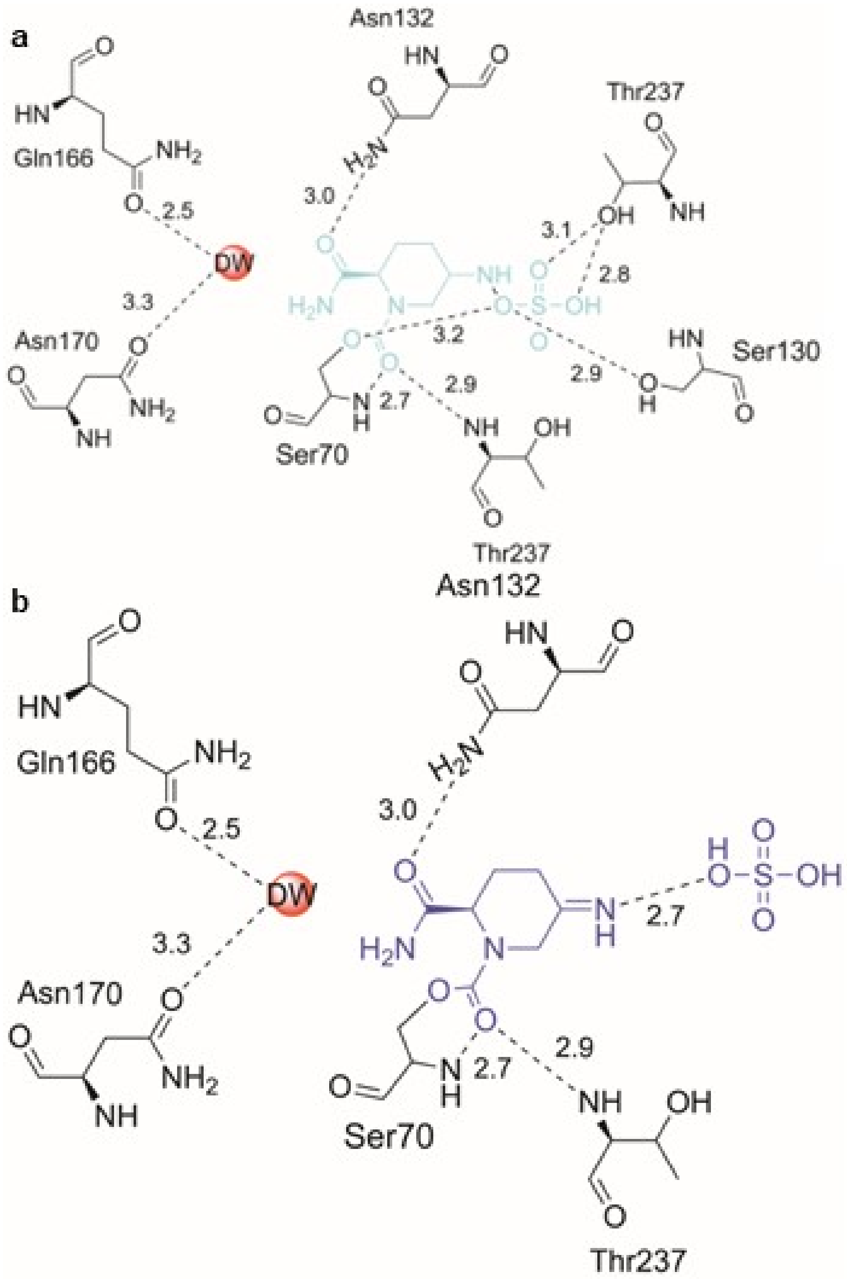

Figure S24: Ligand interactions of the KPC-2^{G89D}:avibactam complex. Hydrogen bonds are shown as dashed lines, distances in Å. The avibactam carbamyl is coloured cyan (with sulphate present, a) or purple (desulphated, b).

SUPPLEMENTARY INFORMATION REFERENCES

1. Oliveira, A.S.F., et al., *Dynamical nonequilibrium molecular dynamics reveals the structural basis for allostery and signal propagation in biomolecular systems.* The European Physical Journal B, 2021. **94**(7): p. 144.
2. Liakopoulos, A., D. Mevius, and D. Ceccarelli, *A Review of SHV Extended-Spectrum β-Lactamases: Neglected Yet Ubiquitous.* Frontiers in Microbiology, 2016. **7**.
3. Poirel, L., et al., *Emergence in Klebsiella pneumoniae of a chromosome-encoded SHV beta-lactamase that compromises the efficacy of imipenem.* Antimicrobial agents and chemotherapy, 2003. **47**(2): p. 755-758.
4. Rodkey, E.A., et al., *Crystal Structure of a Preacylation Complex of the β-Lactamase Inhibitor Sulbactam Bound to a Sulfenamide Bond-Containing Thiol-β-lactamase.* Journal of the American Chemical Society, 2012. **134**(40): p. 16798-16804.
5. Pettersen, E.F., et al., *UCSF Chimera—A visualization system for exploratory research and analysis.* Journal of Computational Chemistry, 2004. **25**(13): p. 1605-1612.
6. Mirdita, M., et al., *ColabFold: making protein folding accessible to all.* Nature Methods, 2022. **19**(6): p. 679-682.
7. Pemberton, O.A., et al., *Heteroaryl Phosphonates as Noncovalent Inhibitors of Both Serine- and Metallocarbapenemases.* Journal of Medicinal Chemistry, 2019. **62**(18): p. 8480-8496.
8. Laskowski, R.A. and M.B. Swindells, *LigPlot+: Multiple Ligand–Protein Interaction Diagrams for Drug Discovery.* Journal of Chemical Information and Modeling, 2011. **51**(10): p. 2778-2786.
9. Tooke, C.L., et al., *Natural variants modify Klebsiella pneumoniae carbapenemase (KPC) acyl–enzyme conformational dynamics to extend antibiotic resistance.* Journal of Biological Chemistry, 2021. **296**: p. 100126.
10. Tooke, C.L., et al., *Tautomer-Specific Deacylation and Ω-Loop Flexibility Explain the Carbapenem-Hydrolyzing Broad-Spectrum Activity of the KPC-2 β-Lactamase.* Journal of the American Chemical Society, 2023. **145**(13): p. 7166-7180.
11. Krishnan, N.P., et al., *Inhibition of Klebsiella β-Lactamases (SHV-1 and KPC-2) by Avibactam: A Structural Study.* PLOS ONE, 2015. **10**(9): p. e0136813.
